# Oscillating the local milieu of polymersome interiors via single input-regulated bilayer crosslinking and permeability tuning

**DOI:** 10.1038/s41467-022-28227-6

**Published:** 2022-01-31

**Authors:** Guhuan Liu, Jiajia Tan, Jie Cen, Guoying Zhang, Jinming Hu, Shiyong Liu

**Affiliations:** CAS Key Laboratory of Soft Matter Chemistry, Department of Polymer Science and Engineering, School of Chemistry and Materials Science, University of Science and Technology of China, 230026 Hefei, Anhui China

**Keywords:** Bioinspired materials, Supramolecular polymers, Biomaterials

## Abstract

The unique permselectivity of cellular membranes is of crucial importance to maintain intracellular homeostasis while adapting to microenvironmental changes. Although liposomes and polymersomes have been widely engineered to mimic microstructures and functions of cells, it still remains a considerable challenge to synergize the stability and permeability of artificial cells and to imitate local milieu fluctuations. Herein, we report concurrent crosslinking and permeabilizing of pH-responsive polymersomes containing Schiff base moieties within bilayer membranes via enzyme-catalyzed acid production. Notably, this synergistic crosslinking and permeabilizing strategy allows tuning of the mesh sizes of the crosslinked bilayers with subnanometer precision, showing discriminative permeability toward maltooligosaccharides with molecular sizes of ~1.4-2.6 nm. The permselectivity of bilayer membranes enables intravesicular pH oscillation, fueled by a single input of glucose. This intravesicular pH oscillation can further drive the dissipative self-assembly of pH-sensitive dipeptides. Moreover, the permeabilization of polymersomes can be regulated by intracellular pH gradient as well, enabling the controlled release of encapsulated payloads.

## Abstract (web summary)

Permselectivity of cellular membranes is crucial to normal cellular function but this has been hard to replicate in artificial cells. Here, the authors use pH-responsive polymersomes containing Schiff base moieties which can be used to control mesh size allowing for selective permeability control creating responsive systems.

## Introduction

Self-sustained oscillations are ubiquitous in living organisms, and the periodic rhythms are correlated to many biological processes, such as heartbeat, respiration, and cell cycle^1–3^. Remarkably, even at a single-cell level, transient oscillations of intracellular microenvironments are prevalent^4–6^. For instance, intracellular pH oscillation contributes to ion transport, endocytosis, proliferation, apoptosis, and so on^7–12^. To enable these oscillation-related intracellular events, cells have evolved to be highly compartmentalized, in which organelles are enclosed within phospholipid bilayers^13,14^. The permselectivity of phospholipid bilayers only allows the passage of certain ions, nutrients, metabolites, and water molecules, which plays a critical role in maintaining intracellular homeostasis while enabling local transient oscillations^15,16^.

To understand the correlations between intracellular oscillating events and physiological/pathological processes, it is of increasing interest to develop synthetic materials to mimic the structures and functions of cells^17–21^. In this regard, polymersomes (also referred to as polymeric vesicles) represent an ideal candidate due to the similarity of hollow structures to cells that are enclosed with bilayer membranes^22–24^. Unlike liposomes assembled from phospholipids, polymersomes possess increased structural stability yet decreased membrane permeability, which is unfavorable for mimicking intracellular energy exchange and the selective transport of different substances. To this end, several approaches, such as the development of stimuli-responsive vescicles^25–28^, post-modification of bilayer membranes^29–31^, membrane protein insertion^32,33^, have been applied to permeabilize polymersomes. Unfortunately, conventional permeabilizing approaches generally lead to compromised stability and even disruption of polymersome integrity. To resolve this dilemma, our group proposed a “traceless” crosslinking strategy to achieve concurrent stabilizing and permeabilizing bilayers of polymersomes by taking advantage of carbamate linkages as potential primary amine donors^34^. The activation of carbamate linkages allows crosslinking the nanoassemblies through extensive amidation reactions by taking advantage of the in situ generated primary amines, and the resulting amide linkages created numerous hydrophilic channels and endowed the resulting crosslinked bilayers with increased permeability^34–38^. However, despite great achievements in regulating the permeability and stability of polymersomes, the mimicking of intracellular transient oscillations within polymersomes has far less been explored^26,39–42^.

**Fig. 1:**
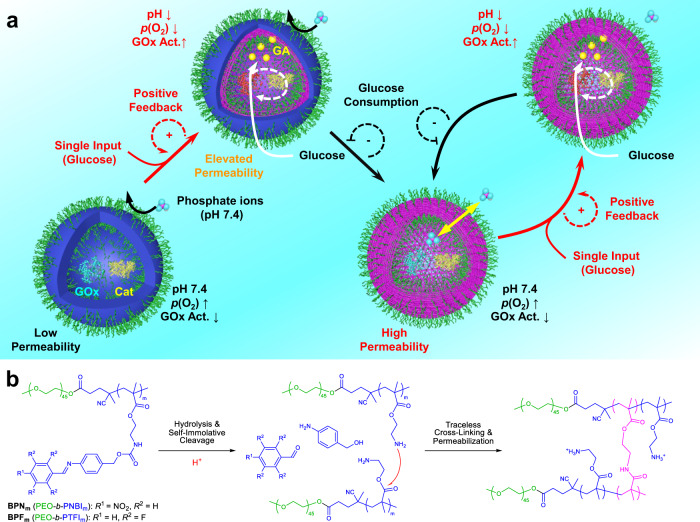
Glucose-fueled self-catalyzed construction of transient acidic milieu within the aqueous lumen of enzyme-loaded polymersomes via self-regulated bilayer permeability. **a** Polymersomes self-assembled from Schiff base-containing amphiphilic block copolymers were loaded with glucose oxidase (GOx) and catalase (Cat). Externally added glucose diffuses across initially hydrophobic vesicle bilayers into the aqueous lumen and GOx/Cat-mediated cascade enzymatic reactions generate gluconic acid (GA), which partially ionizes and leads to lumen pH decrease. The local acidic milieu triggers the hydrolysis of side chain Schiff base moieties within bilayers, leading to subsequent self-immolative degradation, primary amine generation and bilayer crosslinking, and concurrent hydrophobic-to-hydrophilic transition. This process is accompanied by the elevation of glucose permeability through bilayers and enhanced GOx activity due to local acidic lumen. These concurrent events facilitate the construction of a closed positive feedback cycle in a self-catalyzed manner toward enzymatic reaction rates and lumen pH decrease, which further accelerates Schiff base hydrolysis and enhances bilayer permeability. At later stages, external buffer ions and partially ionized GA in the aqueous lumen start to diffuse across newly generated hydrophilic crosslinked bilayers, leading to lumen pH increase and GOx activity decrease. Upon feeding with glucose fuel, we construct transient acidic milieu in the aqueous lumen of vesicles by coordinating cascade enzymatic reactions with bilayer permeability. By finely tuning the extent of bilayer crosslinking and corresponding mesh sizes, pH oscillation in the aqueous lumen could be established via the addition of multiple glucose dosages. **b** Chemical structures of amphiphilic block copolymers, **BPN** and **BPF**, and mechanisms of acidic pH-triggered Schiff base hydrolysis, self-immolative cleavage, amidation-mediated bilayer crosslinking, and regulated vesicle bilayer permeabilization.

Herein, we report the self-regulated intravesicular oscillations of local pH via the coupling of positive and negative feedback loops, mimicking transient intracellular acidification (Fig. 1). Specifically, glucose oxidase (GOx) and catalase (Cat) are loaded into pH-responsive polymersomes self-assembled from Schiff base-containing amphiphilic block copolymers (BCPs). Upon dispersing the polymersomes in phosphate buffer (PB) containing glucose, the diffusion of glucose and phosphate ions into polymersome interiors is initially inhibited due to the low permeability of bilayer membranes. However, the hydrolysis of the labile imine linkages results in gradually increased permeability of bilayer membranes via traceless crosslinking, enabling enhanced passage of glucose but retarded diffusion of phosphate ions into the aqueous lumens of polymersomes. The tandem enzyme-catalyzed reactions are then activated, catalyzing the conversion of glucose into gluconic acid (GA). The locally generated GA thus, in turn, increases GOx activity, facilitating imine hydrolysis, and further permeabilizing the bilayers in a positive feedback loop. Notably, the accumulation of GA results in local acidification within polymersome lumens until the depletion of glucose and the diffusion of phosphate ions to recover the pH, enabling local transient pH oscillations within polymersome interiors (Fig. 1).

## Results

### Synthesis and self-assembly of pH-responsive Schiff base-containing BCPs

Schiff bases undergo spontaneous hydrolysis at acidic pH, and the pH-responsive properties can be finely tuned by the substituting groups^43–45^. To fabricate pH-responsive BCPs, *p*-nitrobenzaldehyde and 2,3,5,6-tetrafluorobenzaldehyde were modified with *p*-aminobenzyl alcohol with the formation of NBIA and TFIA precursors, followed by the functionalization of hydroxyl groups with 2-isocyanatoethyl methacrylate. The formation of NBIA and TFIA precursors and the corresponding imine-based monomers (NBI and TFI) were characterized by both nuclear magnetic resonance (NMR) and high-resolution mass spectroscopy (HRMS) (Supplementary Figs. 1-5). Reversible addition-fragmentation chain transfer (RAFT) polymerization of NBI and TFI monomers using PEG_45_-based macroRAFT agent (PEG_45_-CTA) afforded pH-responsive PEG_45_-*b*-PNBI_m_ (**BPN**_**m**_, m = 8, 23, 36, and 40) and PEG_45_-*b*-PTFI_m_ (**BPF**_**m**_, m = 6, 25, and 35) diblock copolymers (Supplementary Fig. 6, and Table 1). Self-assembling of these BCPs was conducted by slowly adding water into **BPN**_**m**_ and **BPF**_**m**_ solutions in 1,4-dioxane, respectively. By varying the chemical structures, compositions, and hydrophobic block lengths, nanoassemblies with uniform morphologies including spherical micelles, vesicles, and large compound vesicles (LCVs) were obtained. These nanoassemblies were characterized by dynamic light scattering (DLS), (cyrogenic) transmission electron microscopy ((cryo)-TEM), and scanning electron microscopy (SEM) (Supplementary Figs. 7-9). The structural parameters of the resulting BCPs and corresponding nanostructures are summarized in Table 1.

**Table 1:** Structural parameters of acidic milieu-reactive amphiphilic diblock copolymers and corresponding morphologies of self-assembled nanostructures in aqueous media.

Entries	Diblock copolymers	*M*_n,NMR_ (kDa)^a^	*M*_n,GPC_ (kDa)^b^	*M*_w_/*M*_n_^b^	Aggregates morphology^c^	<*D*_h_ > (nm)^d^
**BPN**_**8**_	PEO_45_-*b*-PNBI_8_	5.6	21.7	1.23	M	70
**BPN**_**23**_	PEO_45_-*b*-PNBI_23_	11.4	33.9	1.18	V	750
**BPN**_**36**_	PEO_45_-*b*-PNBI_36_	16.8	37.5	1.21	V & LCV	1150
**BPN**_**40**_	PEO_45_-*b*-PNBI_40_	18.4	41.5	1.20	LCV	1760
**BPN**_**21**_**-***NBD*	PEO_45_-*b*-P(NBI_0.99_-*co*-NBD_0.01_)_21_	10.7	32.8	1.12	V	690
**BPN**_**24**_**-***NR*	PEO_45_-*b*-P(NBI_0.99_-*co*-NR_0.01_)_24_	11.9	34.2	1.10	V	720
**BPF**_**6**_	PEO_45_-*b*-PTFI_6_	4.6	18.3	1.21	M	50
**BPF**_**25**_	PEO_45_-*b*-PTFI_25_	13.0	49.4	1.13	V	720
**BPF**_**35**_	PEO_45_-*b*-PTFI_35_	17.3	50.3	1.16	V & LCV	1170
**BPF**_**25**_**-***NBD*	PEO_45_-*b*-(TFI_0.99_-*co*-NBD_0.01_)_23_	12.1	35.8	1.20	V	690
**BPF**_**25**_**-***NR*	PEO_45_-*b*-(TFI_0.99_-*co*-NR_0.01_)_24_	12.5	36.2	1.28	V	700

^a^Determined by ^1^H NMR analysis.

^b^Number-average molecular weights, *M*_n_, and molecular weight distributions, *M*_w_/*M*_n_, determined by GPC using DMF as eluent (1.0 mL/min).

^c^Determined by TEM and SEM analysis (M = micelles, V = vesicles, LCV = large compound vesicles).

^d^Determined by DLS measurements.

### Acidic pH-triggered imine hydrolysis

**Fig. 2:**
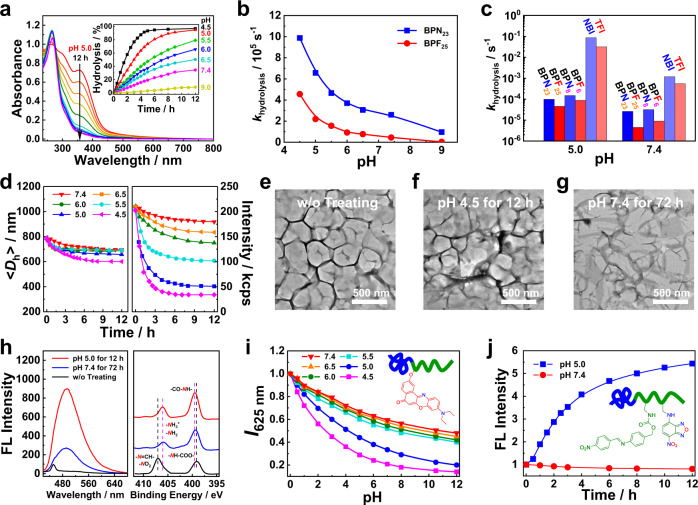
pH-regulated imine hydrolysis, self-immolative cleavage, bilayer crosslinking, and membrane permeabilization. **a** Time-dependent evolution of UV-Vis absorbance spectra and hydrolysis kinetics of imine linkages (inset) recorded for aqueous dispersions of **BPN**_**23**_ polymersomes (0.2 g/L) at varying pH. **b** pH-dependent variation and **c** summary of hydrolysis rate constants, *k*_hydrolysis_, of imine linkages determined for aqueous dispersions of **BPN** and **BPF** polymersomes (~400 μM imine linkages); *k*_hydrolysis_ values of small molecule **NBI** and **TFI** (400 μM in DMSO/water, 3/7 v/v) are included for comparison. **d** Time-dependent evolution of scattered light intensities (left) and intensity-average hydrodynamic diameters, <*D*_h_ > (right), recorded for **BPN**_**23**_ polymersome dispersion (0.2 g/L) at varying pH. **e**–**g** TEM images (scale bars: 500 nm) recorded for **BPN**_**23**_ polymersomes (0.2 g/L) before (**e**) and after incubating at pH 5.0 for 12 h (**f**) and pH 7.4 for 72 h (**g**). **h**, left, Fluorescence emission spectra (*λ*_ex_ = 390 nm) recorded for **BPN**_**23**_ polymersome dispersion upon incubating at pH 5.0 for 12 h and pH 7.4 for 72 h, followed by reacting with fluorescamine (FA) for 15 min in PBS buffer (pH 7.4). **h**, right, XPS N1s spectra of lyophilized **BPN**_**23**_ polymersome dispersions (0.2 g/L) after hydrolysis at pH 5.0 for 12 h and pH 7.4 for 72 h. **i**, **j** Time-dependent evolution of emission intensities recorded for **i** **BPN**_**24**_**-***NR* (*λ*_ex_ = 550 nm, *λ*_em_ = 625 nm) and **j** **BPN**_**21**_**-***NBD* (*λ*_ex_ = 480 nm, λ_em_ = 550 nm) polymersome dispersions (0.2 g/L) in the pH range of 4.5–7.4.

The pH-regulated hydrolysis of imine linkages was first studied. Taking **BPN**_**23**_ polymersomes as an example, less than 10% imine hydrolysis was observed after 36 h incubation at pH 9.0, as evidenced by UV-vis spectra (Supplementary Fig. 10). However, the hydrolysis extents gradually increased upon lowing the solution pH. Specifically, at pH 5.0, the absorbance peak of **BPN**_**23**_ vesicles centered at ∼356 nm dramatically decreased, and ~95% imine moieties were hydrolyzed within ∼8 h (Fig. 2a). Notably, at pH 7.4, ~30% of imine bonds were hydrolyzed within 12 h, which was further increased to ∼80% after 96 h. The hydrolysis process followed a pseudo-first-order kinetics model with a half-life (*t*_1/2_) of ~21 h at pH 7.4 (Supplementary Fig. 11a). Besides acidic pH, the substituting groups on the benzaldehyde precursors can directly influence the stability of the imine linkages, and **BPF**_**25**_ polymersomes generally had slower hydrolysis rates than that of **BPN**_**23**_ at identical pH (Fig. 2b and Supplementary Fig. 11b). To elucidate the effect of the formation of nanoassemblies on pH-mediated hydrolysis of imines, we examined the hydrolysis behavior of **NBI** and **TFI** monomers at pH 5.0 and 7.4 as well. Incubation of **NBI** and **TFI** monomers at pH 5.0 led to very fast hydrolysis, and a complete conversion was achieved within ~1 min. Even at pH 7.4, the hydrolysis of **NBI** and **TFI** monomers reached a plateau after ~90 min incubation (Supplementary Fig. 12). Therefore, the formation of nanoassemblies greatly inhibited the imine hydrolysis process, while the hydrolysis rates were further retarded by increasing the block lengths of the imine-containing blocks (Fig. 2c, Supplementary Fig. 13). This observation was attributable to the suppressed diffusion of protons into the initially hydrophobic bilayers of polymersomes or micellar cores.

### Acidic pH-triggered concurrent crosslinking and permeablizing of polymersome bilayers

Next, we examined the pH-responsive properties of **BPN**_**23**_ and **BPF**_**25**_ polymersomes by DLS. At pH 9.0, **BPN**_**23**_ vesicle dispersion exhibited an intensity-average hydrodynamic diameter, <*D*_h_ > , of ∼800 nm and a polydispersity index (*μ*_2_/*Γ*^2^) of ∼0.208. Upon pH drop from 9 to 7.4, the scattering intensity exhibited ~10% decrease whilst <*D*_h_ > decreased to 695 nm after 12 h incubation. By contrast, the <*D*_h_ > was dropped to ~680 nm after 12 h incubation, while the corresponding scattering light intensities showed a 75% decrease at pH 5.0 (Fig. 2d). Similar changes in the scattering intensities and <*D*_h_ > were observed for **BPF**_**25**_ polymersomes (Supplementary Fig. 14). Moreover, the negligible changes in <*D*_h_ > yet an evident decrease of the scattering intensities were also observed for **BPN**_**8**_ and **BPF**_**6**_ micellar nanoparticles under otherwise identical conditions (Supplementary Fig. 15).

For both **BPN**_**23**_ and **BPF**_**25**_ polymersomes, vesicular nanostructures were well retained at both pH 5.0 and 7.4, as observed by TEM and SEM (Fig. 2e–g, Supplementary Figs. 14 and 16). These results were consistent with the DLS measurements that indicated the presence of nanoassemblies after incubation at pH 5.0 and 7.4 (Fig. 2d). Notably, **BPN**_**23**_ polymersomes after incubation at pH 5.0 for 12 h and pH 7.4 for 72 h cannot disintegrate into unimers anymore even in the presence of an excessive amount of dimethyl sulfoxide (DMSO), revealing the formation of crosslinked nanostructures after hydrolysis (Supplementary Fig. 16c).

As suggested in our previous reports^34,37^, carbamate linkages could be potentially used as primary amine donors that can undergo inter/intrachain amidation reactions once being decaged upon activation by external stimuli. In the current case, we envisioned that the hydrolysis of imine linkages led to the formation of 1,4-benzoquinoneimine intermediate through self-immolative 1,6-elimination reactions and the release of primary amines. The in situ formed primary amines underwent either protonation or inter/intrachain amidation reactions. The presence of primary amines within the crosslinked bilayers was confirmed by the fluorescamine (FA) probe, which was initially nonfluorescent yet highly fluorescent after reacting with primary amines. The higher FA emission of polymersomes obtained at pH 5.0 for 12 h implied that more primary amines were generated than that at pH 7.4 for 72 h (Fig. 2h). On the other hand, the depletion of ester bonds with the formation of amide linkages within **BPN**_**23**_ polymersome bilayers after hydrolysis was verified by X-ray photoelectron spectroscopy (XPS), Fourier-transform infrared (FT-IR) spectroscopy, and ^1^H NMR (Fig. 2h and Supplementary Fig. 16). Quantitative analysis of XPS core-level N1s spectra revealed that, after the hydrolysis of imine bonds, the residual contents of protonated amine species were ∼32% and ~19% of all *N*-relevant species within the final crosslinked polymersomes, respectively (Fig. 2h). FT-IR spectra revealed that a higher content of amide bonds was formed at pH 7.4 than that of pH 5.0 (Supplementary Fig. 16d). Collectively, the ratio of protonated and non-protonated amines within the hydrolyzed polymersomes was highly dependent on the solution pH, and a lower solution pH led to increased protonated amines yet decreased amide formation. Because the protonated amines did not contribute to crosslinking and only the non-protonated amines implemented intra/interchain amidation reactions, the decreased solution pH thus resulted in a lower crosslinking density^34,37^. Therefore, the crosslinking density of bilayer membranes can be readily tuned by solution pH.

To further confirm that concurrent crosslinking and polarity switching have indeed occurred within bilayer membranes after imine hydrolysis, we incorporated a polarity-sensitive probe, Nile red (NR), into the pH-responsive polymersomes. NR-labeled BPs of **BPN**_**24**_**-***NR* and **BPF**_**25**_-*NR* were successfully synthesized (Supplementary Fig. 1). Upon incubation of **BPN**_**24**_**-***NR* and **BPF**_**25**_-*NR* polymersomes at varying pH (e.g., 4.5–7.4), a continuous decrease of NR emission intensities at 625 nm was observed, indicating that the bilayer membranes experienced a hydrophobic-to-hydrophilic transition subjected to imine hydrolysis (Fig. 2i and Supplementary Fig. 17). In addition, 4-nitro-2,1,3-benzoxadiazole (NBD)-labeled BP of **BPN**_**21**_**-***NBD* was also synthesized, and the fluorescence of NBD was initially quenched after self-assembling into polymersomes. Upon incubating **BPN**_**21**_**-***NBD* polymersomes at pH 4.5, a 5.4-fold fluorescence increase was observed within 12 h, whereas no significant fluorescence changes were detected at pH 7.4 (Fig. 2j and Supplementary Fig. 18). This result was likely ascribed to the different hydrolysis extents of imine bonds at varying pH (95% at pH 5.0 vs 40% at pH 7.4). Interestingly, there was a linear relationship between the NBD fluorescence and imine hydrolysis extents, rendering it possible to monitor the hydrolysis degree of imine linkages by fluorescence changes (Supplementary Fig. 18d). By contrast, the fluorescence of NBD cannot be quenched within **BPF**_**25**_**-***NBD* polymersomes, revealing negligible fluorescence changes upon imine hydrolysis (Supplementary Fig. 19). Building on the above results, we assumed that the presence of nitrobenzene moieties accounted for the fluorescence quenching of NBD within **BPN**_**21**_**-***NBD* polymersomes^46,47^.

### Acidic pH-regulated permselectivity of polymersome bilayers

**Fig. 3:**
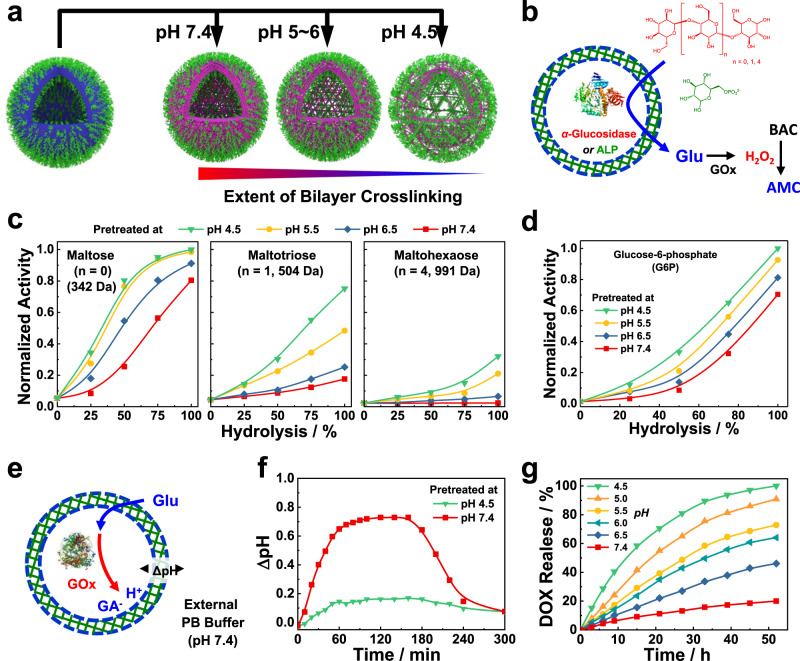
pH-regulated polymersome bilayer permselectivity. **a** Schematics of modulating the extent of bilayer crosslinking and corresponding mesh sizes via pH adjustment. **b** Schematics of assaying bilayer permeability by coupling enzymatic reactions inside aqueous interiors of polymersomes with externally added substrates of varying molecular sizes and charges. The aqueous lumen is loaded with either *α*-glucosidase or alkaline phosphatase (ALP). **c**, **d** Variation of normalized activities (i.e., initial rates of H_2_O_2_-actuated fluorogenic transformation of BAC into AMC) as a function of hydrolysis extents of imine linkages in **c** *α*-glucosidase-loaded and **d** ALP-loaded **BPN**_**23**_ polymersomes (0.2 g/L); the polymersome dispersions were pretreated at varying pH to reach specified extents of imine hydrolysis and then adjusted to pH 7.4 using PB buffer. Into the external buffer media, GOx (1.0 mg/L), BAC (50 μM), and one of the corresponding enzymatic substrates including maltose, maltotriose, maltohexose, and glucose 6-phosphate (G6P) were added, the evolution of fluorogenic AMC emission was then monitored for 30 min. **e** Schematics of the construction of transient acidic milieu inside the aqueous lumen of **BPN**_**23**_ polymersomes (0.2 g/L) encapsulating GOx using externally added glucose as substrate. **f** Evolution of pH discrepancy, ΔpH, between the aqueous lumen and external media (PB buffer; pH 7.4) upon addition of 2 mM glucose. GOx-loaded **BPN**_**23**_ polymersomes (0.2 g/L) were pretreated at pH 4.5 for 10 h and pH 7.4 for 100 h, respectively, and then adjusted back to pH 7.4 using PB buffer (30 mM). **g** Release profiles of DOX from drug-loaded **BPN**_**23**_ polymersomes (0.2 g/L) upon incubation at varying pH.

As detailed above, the crosslinking density of bilayer membranes can be delicately tuned by solution pH, and a decreased pH value led to a faster imine hydrolysis rate but a lower crosslinking density (Fig. 3a). To probe the mesh sizes of the bilayer membranes of crosslinked vesicles obtained at varying pH, we used maltooligosaccharides with different molecular weights (maltose, 342 Da; maltotriose, 504 Da; maltohexaose, 991 Da) and negatively charged glucose 6-phosphate (G6P, 258 Da) as enzymatic substrates. Specifically, norbornene-modified α-glucosidase or alkaline phosphatase (ALP) was loaded into the aqueous lumens of **BPN**_**23**_ polymersomes. The unloaded enzymes were removed by passing an enzyme-affinity column filled with tetrazine-conjugated Sephadex G-20 beads by taking advantage of high-efficiency inverse electron-demand Diels-Alder (IEDDA) reactions between norbornene and tetrazine (Supplementary Fig. 20)^48^. Moreover, the Nor-modified enzymes only showed a slightly decreased activity (~80–92%) compared to the intact unmodified enzymes (Supplementary Fig. 21). Note that the diffusion of maltooligosaccharides or G6P into the polymersome lumens led to the production of glucose under enzyme-catalyzed hydrolysis. The generated glucose was further converted to hydrogen peroxide (H_2_O_2_) in the presence of glucose oxidase (GOx). Moreover, the H_2_O_2_ contents can be detected by the conversion of nonfluorescent 4-(4,4,5,5-tetramethyl-1,3,2-dioxaborolan-2-yl)benzyl (4-methyl-2-oxo-2H-chromen-7-yl)carbamate (BAC) to highly fluorescent 7-amino-4-methylcoumarin (AMC) (Fig. 3b)^49,50^. Therefore, the diffusion of maltooligosaccharides or G6P across the crosslinked bilayer membranes can be indirectly reflected by the fluorescence changes of AMC.

As indicated by the fluorescence signals, the diffusion of all three maltooligosaccharides and G6P across the crosslinked bilayers was fully restrained before imine hydrolysis because of the low permeability of bilayer membranes. Upon increasing the hydrolysis degrees of imine linkages, the permeability of crosslinked membranes gradually elevated and hydrophilic network channels were generated, enabling the passage of maltooligosaccharides and G6P into polymersome interiors (Fig. 3c and 3d). For maltohexaose (~2.6 nm in diameter; Supplementary Fig. 22), even if the imine linkages were thoroughly hydrolyzed at pH 7.4, the diffusion across the vesicular bilayers was completely suppressed, whereas maltose (~1.4 nm in diameter) can diffuse across the vesicular bilayers at the same conditions. The fluorescence changes upon maltotriose (~1.7 nm in diameter) addition were in between maltose and maltohexaose (Fig. 3c). Notably, despite being negatively charged, the diffusion of G6P was not remarkably affected even after polymersome hydrolysis at pH 7.4, which could be ascribed to the low molecular weight (258 Da) (Fig. 3d). Taken together, the pH-mediated crosslinking and permeabilizing of the bilayer membranes enabled the passage of molecules with specific molecular weight cutoff. More importantly, the crosslinking density and corresponding mesh sizes can be readily tuned by solution pH with subnanometer precision. However, it was rather challenging to modulate the permeability of polymersomes with molecular size precision in previous studies^22^.

Similar to the selective permeability of cell membranes, the unprecedented permselectivity of the resulting crosslinked polymersomes only allowed the passage of molecules with specific dimensions and rendered it possible to manipulate the interior milieu of polymersomes. As a proof-of-example, GOx-loaded **BPN**_**23**_ polymersomes were pre-incubated at pH 7.4 and 5.0 to obtain vesicles with exhaustive imine hydrolysis. After the addition of glucose (180 Da; 2 mM) solution in PB buffer (30 mM) into the polymersome dispersions, glucose gradually diffused into the polymersome lumens, which were further converted to GA in the presence of GOx, lowing intravesicular pH values. On the other hand, the negatively charged phosphate ions with a slower diffusion rate than that of glucose then entered the aqueous lumens of polymersomes to compensate for the pH decrease, resulting in transient pH oscillations (Fig. 3e).

To verify the pH-oscillating process, the inner pH of the crosslinked vesicles was monitored by 8-hydroxypyrene-1,3,6-trisulfonic acid trisodium salt (HPTS) (Supplementary Fig. 23)^51^, which was retained within the aqueous lumens of polymersomes even after imine hydrolysis due to the presence of negative charges (Supplementary Fig. 24). The pH discrepancy within (pH_in_) and outside (pH_out_) the crosslinked polymersomes (ΔpH = pH_out_ − pH_in_) was then examined, revealing a bell-shaped curve upon glucose addition (Fig. 3f). For **BPN**_**23**_ polymersomes pretreated at pH 7.4, ΔpH gradually increased and stabilized at ~0.7 after 60 min until the glucose was consumed, which finally returned to ~0.1 after 300 min incubation. By contrast, ΔpH was maintained at ~0.1 for the same vesicles pretreated at pH 4.5 (Fig. 3f). This result revealed the distinct permeability of crosslinked bilayer membranes toward glucose and phosphate ions, and this unique permselectivity allowed local transient pH oscillations within the crosslinked polymersomes.

Notably, the pH-triggered permeabilization of the vesicles can also be used for the release of encapsulated payloads. Using doxorubicin hydrochloride (DOX) as an example, the release profiles of DOX-loaded **BPN**_**23**_ and **BPF**_**25**_ polymersomes were investigated. As shown in Fig. 3g, less than 20% DOX was released from **BPN**_**23**_ polymersomes upon incubation at pH 7.4 after 52 h. Upon lowering the solution pH, increased DOX release amounts were observed. Specifically, ~100% cumulative DOX was obtained after 52 h incubation at pH 4.5. Due to a slower hydrolysis rate of imine linkages, DOX released from **BPF**_**25**_ vesicles was slower than that of **BPN**_**23**_ polymersomes. For example, 98% DOX was released from DOX-loaded **BPF**_**25**_ polymersomes after ~130 h incubation at pH 4.5, whereas only 10% DOX was released within the same period at pH 7.4 (Supplementary Fig. 25).

### Intravesicular pH oscillations via glucose-regulated bilayer crosslinking and permeabilization

**Fig. 4:**
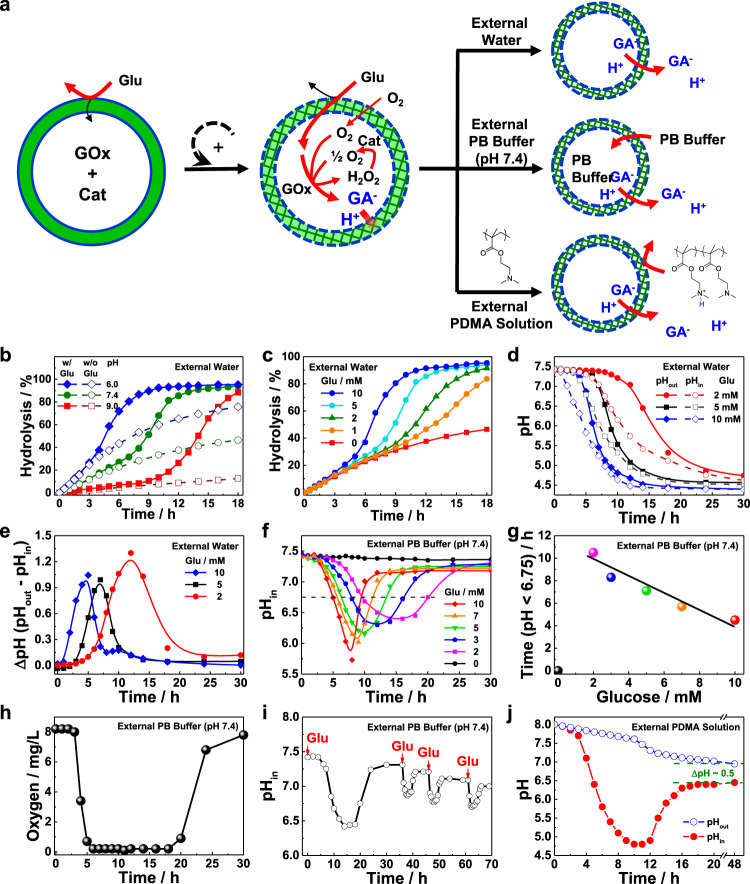
Construction of local transient acidic milieu and pH oscillation within polymersome interiors. **a** Schematics of pH-oscillating nanosystems constructed from **BPN** polymersomes encapsulating glucose oxidase (GOx) and catalase (Cat) in varying external media including pure water, PB buffer (pH 7.4), and an aqueous solution of cationic polyelectrolyte (PDMA). Incubation time-dependent evolution of hydrolysis extents of imine linkages (**b**, **c**), local pH of polymersome interior, pH_in_, and external pH, pH_out_ (**d**), and pH discrepancy, ΔpH, between inside and outside milieu (**e**) recorded for **BPN**_**23**_ polymersomes encapsulating GOx and Cat enzymes in the absence and presence of glucose (**b**, 5 mM; **c–e**, 0~10 mM), with the initial external milieu set at pH 6.0–9.0 (**b**) and pH 7.4 (**c–e**), respectively. Incubation time-dependent evolution of pH_in_ (**f**) and summary of time durations with pH_in_ < 6.75 at varying glucose concentrations (**g**) recorded for **BPN**_**23**_ polymersomes encapsulating GOx and Cat enzymes in the presence of external PB buffer (pH 7.4, 30 mM). **h** Evolution of local oxygen levels within the aqueous interior of **BPN**_**23**_ polymersomes encapsulating [Ru(dpp)_3_]Cl_2_, GOx, and Cat in the presence of external PB buffer (pH 7.4, 30 mM) upon addition of 5 mM glucose. **i** Time-dependent pH_in_ oscillations recorded for **BPN**_**23**_ polymersomes encapsulating GOx and Cat in the presence of external PB buffer (pH 7.4, 30 mM), and 2 mM glucose was introduced at specified time intervals. **j** Evolution of pH_in_ and pH_out_ recorded for **BPN**_**23**_ polymersomes encapsulating GOx and Cat in the presence of external aqueous PDMA medium (1.57 g/L, ~10 mM tertiary amine moieties) upon addition of 5 mM glucose.

Considering that GOx-mediated catalysis of glucose leads to the generation of acidic GA, the generated GA can be used to in situ actuate the imine hydrolysis as well. Glucose-fueled hydrolysis of imine linkages of **BPN**_**23**_ polymersomes was then investigated. GOx and Cat were encapsulated into the aqueous lumens of **BPN**_**23**_ polymersomes. The vesicle dispersion was initially adjusted to pH 7.4, and the GOx was less active at this pH (Supplementary Fig. 26). The hydrolysis of imine bonds was monitored by UV-vis spectra in the presence of glucose (5 mM). Although a higher pH (pH 6.0, 7.4, and 9.0) led to increased induction periods, the addition of glucose remarkably elevated the hydrolysis rates once the induction period was completed compared to that of the glucose-free systems at all tested pH (Fig. 4b). The accelerated hydrolysis rates were ascribed to the increased production of GA under cascade enzyme catalysis in the presence of GOx and Cat, and the decreased pH and increased activity of GOx further boosted the hydrolysis of imine bonds in a positive feedback manner. Moreover, this self-amplification process can also be reported by the fluorescence turn-on of NBD using GOx/Cat@**BPN**_**21**_**-***NBD* polymersomes, revealing a faster fluorescence increase in the presence of higher glucose concentrations (Supplementary Fig. 27).

In addition, we found that the hydrolysis rates could also be tuned by the glucose concentrations at a fixed pH of 7.4, suggesting that a higher glucose concentration led to a shorter induction period yet faster hydrolysis rate. Specifically, the induction period dropped from 10 to 5 h, and the complete hydrolysis of imine bonds decreased from more than 18 to 11 h upon adding 0–10 mM glucose (Fig. 4c). We monitored the pH_in_ and pH_out_ of the polymersomes using the HPTS probe and a pH meter, respectively. Both pH_in_ and pH_out_ experienced continuous drops upon glucose addition, while pH_in_ was constantly lower than that of pH_out_ until they became identical after the depletion of glucose (Fig. 4d). The asynchrony of pH changes produced a transmembrane pH gradient, reaching maximum values of 1-1.3 upon the addition of 2-10 mM glucose (Fig. 4e).

To compensate for the produced GA, the polymersomes were dispersed in PB buffer instead of deionized water, and the pH_in_ was expected to be restored after GA was neutralized. We found that the polymersomes were relatively stable even in the presence of 50 mM PB buffer, as evidenced by the negligible changes in hydrodynamic diameters (Supplementary Fig. 28). We subsequently monitored pH_in_ changes of GOx/Cat@**BPN**_**23**_ polymersomes (pH 7.4) in the presence of varying concentrations of glucose (0–10 mM). The entry of phosphate ions was initially blocked before imine hydrolysis and decreased pH_in_ was observed due to GA generation. Upon extending the incubation time, the pH_in_ was gradually recovered to initial values due to the increased permeability of bilayer membranes and the entry of phosphate ions. Moreover, a higher glucose concentration led to a lower pH_in_ and a shorter recovery time (Fig. 4f). We arbitrarily chose pH_in_ < 6.75 to describe the lasting time of the pH oscillation process and found that the lasting time was linearly correlated with the glucose concentrations with a negative slope between 2 and 10 mM glucose (Fig. 4g). Notably, this unique inverse sensitivity can be potentially used for highly sensitive pH detection^52,53^.

In addition to intravesicular pH oscillation, a local fluctuation of oxygen (O_2_) concentration was also achieved due to the net O_2_ consumption in GOx/Cat cascade enzyme-catalyzed reactions. The evolution of O_2_ concentration was monitored by encapsulating an O_2_-sensitive probe of [Ru(dpp)_3_]Cl_2_ into the polymersome lumens (Fig. 4h). The phosphorescence intensity of [Ru(dpp)_3_]Cl_2_ was highly dependent on the O_2_ concentrations, and a linear quenching behavior was observed at the O_2_ concentrations of 0–8 mg/L (Supplementary Fig. 29)^54,55^. As such, the O_2_ concentrations can be readily monitored by the phosphorescence intensities. The O_2_ concentration of GOx/Cat@**BPN**_**23**_ polymersomes (external PB buffer, pH 7.4) upon addition of 5 mM glucose decreased from ~8 to 0.2 mg/L after 6 h incubation, which was maintained at 0.2 mg/L between 6 and 18 h, followed by an autonomous recovery to the initial value between 16 and 30 h (Fig. 4h). By contrast, in the absence of polymersomes, the O_2_ concentrations dropped quickly within 0.5 h and gradually recovered to the initial values after 12 h incubation. This result was attributable to the free diffusion of glucose that speeded up the reaction of GOx and glucose in the absence of vesicles. The minimum O_2_ concentration of the GOx/Cat-catalyzed system (4.5 mg/L) was much higher than that of the GOx-catalyzed system (0.2 mg/L) without Cat, while the GOx/Cat-catalyzed system had lower pH values within 5 h (Supplementary Fig. 30). It should be mentioned that the accumulation of H_2_O_2_ under GOx catalysis in the absence of Cat adversely affected the GOx activity, as supported by the decreased O_2_ scavenging capacity after pre-incubating GOx with H_2_O_2_ (Supplementary Fig. 31). Building on the above results, we concluded that it was possible to transiently manipulate the local milieu of polymersome interiors by concurrently crosslinking and permeabilizing bilayer membranes through the traceless crosslinking approach, fueled by the single input of glucose.

After the self-regulated pH oscillation, the imine moieties were hydrolyzed, and the bilayer membranes were crosslinked and permeabilized. Appealingly, repeatable pH oscillations could be achieved as long as the chemical fuel (i.e., glucose) was added, which was ascribed to the distinct diffusion rates of glucose and phosphate ions across the crosslinked membranes (Fig. 4i). Besides PB buffer, cationic poly(2-(dimethylamino)ethyl methacrylate) (PDMA, ~7 kDa) was also used to neutralize GA produced in the cascade enzyme-catalyzed reactions^56^. In striking contrast to water molecules and phosphate ions, PDMA cannot cross the bilayers of **BPN**_**23**_ polymersomes, regardless of before or after imine hydrolysis. Upon glucose addition (5 mM), the minimum pH_in_ of the vesicle decreased to 4.7 and the increased permeabilization of the vesicle bilayers allowed the generated GA within the lumens to diffuse out and to be neutralized by PDMA, elevating pH_in_ while decreasing pH_out_ (Fig. 4j). Unlike the addition of PB buffer with comparable pH_in_ and pH_out_ after 30 h incubation, pH_out_ was higher than that of pH_in_ (ΔpH = 0.5) even after 48 h incubation in the presence of PDMA outside the vesicles. This phenomenon can be explained by Donnan equilibrium due to the impermeable property of the crosslinked bilayer membranes toward PDMA but not GA anions (GA^−^) and protons (H^+^). When reached an equilibrium (H_in_^+^ + GA_in_^−^ = H_out_^+^ + PMDA^+^ + GA_out_^−^), the concentration of GA^−^ ions kept the same within and outside the polymersomes. Therefore, the proton concentration within polymersomes should be higher than that of outside the polymersomes (i.e., H_in_^+^ > H_out_^+^; pH_in_ < pH_out_). This pH gradient across bilayer membranes may be useful to actively load therapeutic agents such as DOX^57,58^.

### Intravesicular pH oscillation-driven dissipative self-assembly of dipeptides

**Fig. 5:**
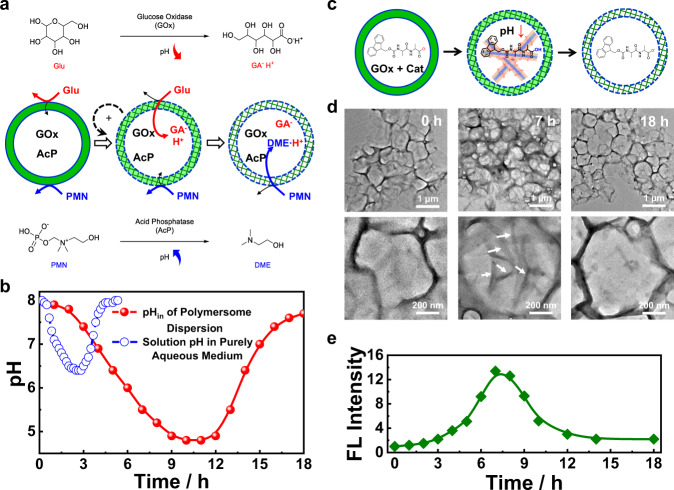
Enzymatically regulated nanosystems for transient dipeptide self-assembly. **a** Schematics of generating transient local acidic milieu within polymersome interiors by kinetically and temporally coordinating two types of enzymatic reactions with opposite pH-tuning directions: GOx converts glucose into gluconic acid (GA), leading to pH decrease, bilayer crosslinking and permeabilization, and enhanced glucose influx (i.e., positive feedback); at later stages, zwitterionic PMN, with higher molar mass compared to glucose, starts to diffuse through bilayers and catalytically generate alkaline DME by acid phosphatase (AcP), leading to pH increase and concomitant enzymatic activity decrease for both GOx and AcP (i.e., negative feedback). **b** Evolution of pH_in_ recorded for **BPN**_**23**_ polymersome dispersion encapsulating GOx and Cat upon addition of glucose (5 mM) and PMN (12 mM); the homogeneous solution mixture containing GOx and Cat was also examined for comparison. The initial pH was both set at 8.0. **c** Feedback-controlled transient acidic milieu of polymersome interiors for temporally programming the self-assembly of Fmoc-AA-OH dipeptide within polymersomes. Evolution of TEM images (**d**) and fluorescence emission intensities (*λ*_ex_ = 405 nm, *λ*_em_ = 480 nm, **e**) recorded for **BPN**_**23**_ polymersomes encapsulating GOx, Cat, Thioflavin T (ThT), and Fmoc-AA-OH dipeptide in the presence of external PB buffer (pH 7.4, 30 mM) upon addition of 10 mM glucose.

To further examine the permselectivity of the pH-responsive polymersomes, besides neutral water molecules, anionic phosphate ions, cationic PDMA polymers, we used zwitterionic ((2-hydroxyethyl)dimethylammonio)methyl hydrogen phosphate (PMN) as a potential base that can be converted to basic 2-dimethylaminoethanol (DME) under acid phosphatase (AcP) catalysis (Supplementary Figs. 32 and 33). At optimal conditions, we chose an enzyme ratio of GOx/AcP = 2/1 (w/w) and the initial concentrations of glucose and PMN were set to 5 and 12 mM, respectively. The starting pH was adjusted to 8.0 and a pH drop from 8.0 to 6.4 was achieved within 3 h in the absence of **BPN**_**23**_ polymersomes, and the pH was further restored to ~8.0 after 6 h incubation following the formation of DME (Fig. 5b). However, in the presence of GOx/AcP@**BPN**_**23**_ polymersomes, we observed that pH_in_ initially dropped to ~4.9 after 10 h incubation, which was lower than that of the vesicle-free system (minimum pH_in_, ~6.4). The pH oscillation period was extended to ~18 h, three times longer than that of the vesicle-free system (Fig. 5b).

In the next phase of work, we attempted to use the intravesicular pH oscillation to drive the dissipative self-assembly of a pH-sensitive dipeptide of Fmoc-Ala-Ala-OH (Fmoc-AA-OH), which was known to form nanofibers at acidic pH but underwent disassembly at basic pH (Supplementary Fig. 34)^59,60^. Fmoc-AA-OH dipeptide, GOx, and Cat were then encapsulated into the aqueous lumens of **BPN**_**23**_ polymersomes. Interestingly, we found that the vesicle formation was not affected by the presence of 18 mg/mL of Fmoc-AA-OH, yet a further increased concentration of Fmoc-AA-OH (e.g., 24 mg/mL) inhibited the formation of **BPN**_**23**_ polymersomes (Supplementary Fig. 35). Moreover, the encapsulation of Fmoc-AA-OH into the polymersome interiors did not adversely affect the formation of nanofibers. The formation of nanofibers within the aqueous lumens and the periphery of polymersomes were observed after incubation at pH 5.0 for 12 h (Supplementary Fig. 36). Upon adding glucose, in the initial stage, the GA generated within the vesicle interiors promoted the protonation of and self-assembly of Fmoc-AA-OH dipeptide (Fig. 5d). Later on, the depletion of glucose and the entry of phosphate ions into the lumens caused the pH recovery, driving the disassembly of the nanofibers (Fig. 5d). The out-of-equilibrium self-assembly process was confirmed by the fluorescence fluctuation of the co-loaded Thioflavin T (ThT) probe^61,62^, exhibiting a strong fluorescence upon binding to nanofibers yet nonfluorescent in a molecularly dissolved state. Despite negligible fluorescence increase within the first 3 h, the fluorescence intensity of ThT increased ~14 times after 7 h incubation, revealing the formation of dipeptide nanofibers. However, the local pH was gradually recovered, thereby disassembling the dipeptide nanofibers and diminishing ThT fluorescence (Fig. 5e).

### Endogenous pH gradient-mediated permeabilization of pH-responsive polymersomes for intracellular drug delivery

pH-responsive nanocarriers in response to endogenous acidic pH have been extensively explored^56,63–67^. To further explore whether the crosslinking and permeabilizing of the bilayer membranes of pH-responsive polymersomes can be operated in living cells by taking advantage of endogenous acidic pH gradients, we probed the permeability changes of bilayer membranes by monitoring the intracellular release of encapsulated chemotherapeutic drug of DOX. To examine the internalization of the pH-responsive polymersomes, we first constructed NBD- and NR-labeled polymersomes through the co-assembly of **BPN**_**21**_**-***NBD*/**BPN**_**24**_**-***NR*, enabling the fabrication of a pH ratiometric probe. Upon incubation at pH 5.0 for 12 h, the fluorescence intensity ratio, *I*_626 nm_/*I*_545 nm_, decreased from 1.7 to 0.4, whereas only little changes were observed at pH 7.4 under otherwise identical conditions (Supplementary Fig. 37). Therefore, polymersomes co-assembled from **BPN**_**21**_**-***NBD*/**BPN**_**24**_**-***NR* can be used for ratiometric detection of acidic pH.

When HepG2 cells were incubated with **BPN**_**21**_-*NBD*/**BPN**_**24**_-*NR* polymersomes, both the red emission of NR and green emission of NBD gradually intensified within 6 h (Supplementary Fig. 38), indicating an efficient internalization of **BPN**_**21**_-*NBD*/**BPN**_**24**_-*NR* polymersomes. Note that the hydrolysis of imine moieties of the internalized polymersomes within endolysosomes led to a further increase of NBD emission but a decrease of NR fluorescence (Supplementary Fig. 37). The colocalization ratios between the NR/LysoTracker blue and NBD/LysoTracker blue gradually increased to ∼88% and ~84% after 6 h incubation, respectively (Supplementary Fig. 38), demonstrating that the internalized **BPN**_**21**_-*NBD*/**BPN**_**24**_-*NR* polymersomes were primarily located within the acidic endolysosomes.

**Fig. 6:**
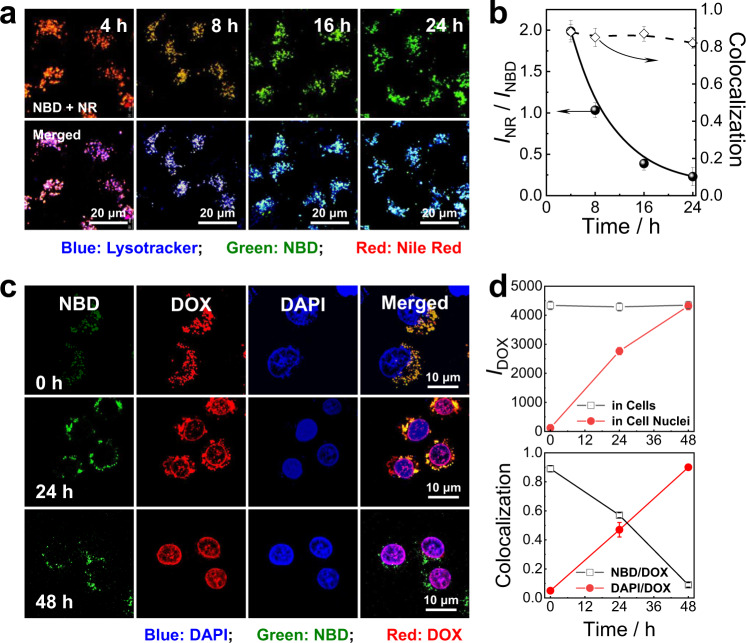
pH-regulated polymersomes as smart nanocarriers within live cells. **a** CLSM images recorded for HepG2 cells. The cells were co-incubated with **BPN**_**21**_-*NBD*/**BPN**_**24**_-*NR* (7/3 wt./wt., 0.2 g/L) co-assembled polymersome dispersion for 6 h; after washing and replacing with fresh culture medium, the cells were further incubated for varying durations (4–24 h) before CLSM experiments. Top: overlay of red and green channel images; bottom: overlay of red, green, and blue channel images. **b** Incubation duration-dependent evolution of emission intensity ratios, *I*_NR_/*I*_NBD_, and colocalization ratios between red channel (*NR*-vesicles) and green channel (*NBD* vesicles) images quantified from CLSM results. Data are presented as mean ± SD (*n* = 3). The endolysosomes were stained with Lysotracker Blue (blue channel). The green channel was excited at 488 nm and collected between 510 and 540 nm; the red channel was excited at 543 nm and collected between 555 and 595 nm; the blue channel was excited at 405 nm and collected between 415 and 475 nm. **c** CLSM images recorded for HepG2 cells. The cells were co-incubated with **BPN**_**21**_**-***NBD* polymersomes (green channel) encapsulating DOX (red channel) in DMEM medium for 6 h; after washing and replacing with fresh DMEM culture medium, the cells were further incubated for varying durations (0–48 h) before CLSM experiments. **b** Incubation duration-dependent normalized DOX emission intensities within whole cells and inside cell nuclei quantified from CLSM results. Error bars represent mean ± SD (*n* = 10 independent cells). **d** Incubation duration-dependent colocalization ratios between red channel (DOX) and green channel (NBD) images, and between red channel (DOX) and blue channel (DAPI, nuclei-staining) images. The blue channel was excited at 405 nm and collected between 450 and 500 nm; the green channel was excited at 488 nm and collected between 510 and 550 nm; the red channel was excited at 543 nm and collected between 590 and 650 nm. Error bars represent mean ± SD (*n* = 10 independent cells).

After 6 h incubation, the non-internalized polymersomes were washed off and the cells were further incubated for another 24 h. During the incubation period, the red fluorescence of NR gradually weakened while the green emission of NBD was intensified (Fig. 6a). The emission ratios of *I*_NR_/*I*_NBD_ quantified from the CLSM images drastically decreased from 2.0 to 0.25 (Fig. 6b), revealing that the acidic pH-triggered concomitant crosslinking and permeabilizing of bilayer membranes of the internalized polymersomes. The colocalization ratios between the green channel of NBD and the blue channel of LysoTracker blue remained constantly high (>80%) throughout the entire incubation period, suggesting that the internalized vesicles were located within the endolysosomes (Fig. 6b).

Since the pH-responsive polymersomes can be efficiently taken up by cancer cells that were retained in acidic organelles such as endolysosomes, the therapeutic performance of DOX-loaded **BPN**_**21**_-*NBD* polymersomes in inhibiting the proliferation of HepG2 cells was then evaluated. After 6 h incubation, the red emission of DOX overlapped quite well with the green emission of **BPN**_**21**_-*NBD* vesicles, and there was no appreciable red emission in the nuclei and cytosols, revealing that DOX was initially located within the aqueous lumens of **BPN**_**21**_-*NBD* polymersomes. However, when the incubation time was extended to 48 h, DOX was gradually released from the polymersomes and eventually entered the nuclei (Fig. 6c). This process was characterized by the decreased colocalization ratios between the green channel of NBD and the red channel of DOX and the concurrently increased colocalization ratios of the blue channel of DAPI and the red channel of released DOX (Fig. 6d). The above results demonstrated the endogenous pH-actuated permeabilizing of bilayers, enabling the release of encapsulated DOX.

In vitro cytotoxicity assay revealed that over 90% of HepG2 cells survived after 48 h incubation with **BPN**_**23**_ and **BPF**_**25**_ polymersomes (up to 1.0 g/L) without DOX loading(Supplementary Fig. 39). Nevertheless, DOX-loaded polymersomes displayed similar toxicity to that of free DOX. Specifically, ~20% of cells survived at an equivalent DOX concentration of 4 μg/mL, and the half-inhibitory concentration (IC_50_) of DOX-loaded polymersomes was determined to be 0.75 μg/mL for **BPN**_**23**_ polymersomes and 0.63 μg/mL for **BPF**_**25**_ polymersomes, respectively. The increased cytotoxicity of DOX-loaded polymersomes over DOX-free polymersomes should be a result of the pH-triggered DOX release within cells.

## Discussion

Polymersomes provide an ideal platform to mimic and investigate the biological functions of cells, having better structural stability yet poor bilayer permeability than liposomes. We previously proposed a traceless crosslinking approach to resolve the dilemma of the stability and permeability of polymersomes using caged carbamate linkages. In this work, pH-responsive imine linkages were used as a new activatable capping agent of carbamate moieties. The hydrolysis rates of imine bonds could be tuned by the substituting groups of the benzaldehydes. The acidic pH-triggered hydrolysis of the imine linkages led to the crosslinking and permeabilizing of the resulting polymersomes. Notably, the crosslinking density of the bilayer membranes could be readily modulated by the solution pH, and a lower pH treatment led to decreased crosslinking density yet increased mesh sizes of the bilayer membranes. More importantly, the resulting crosslinked bilayers conferred selective permeability of varying substances and rendered it possible to achieve intravesicular pH oscillation, fueled by a single input of glucose. To demonstrate the potential applications, we showed the dissipative self-assembly of pH-responsive dipeptides within the polymersome lumens by taking advantage of the intravesicular pH oscillation. Besides external acidic pH, the permeabilization of polymersomes could be also achieved by using endogenous pH gradients, enabling the controlled release of encapsulated payloads for therapeutic purposes.

## Methods

### Sample preparation

Synthetic routes employed for the preparation of NBI and TFI monomers, PEO_45_-*b*-PNBI_n_ (**BPN**_**n**_), PEO_45_-*b*-PTFI_n_ (**BPF**_**n**_), and fluorescent dye-labeled amphiphilic copolymers including **BPN**_**21**_-*NBD*, **BPN**_**24**_-*NR*, **BPF**_**25**_-*NBD*, and **BPF**_**23**_-*NR* are shown in Supplementary Fig. 1.

### Fabrication of polymersome dispersions

Typical procedures used for block copolymer self-assembly are described below. 2.0 mg of the diblock copolymer was dissolved in 1 mL of 1,4-dioxane at 25 °C in a water bath. Then, 2.0 mL of deionized water (pH 9.0) was added within 1 h using a syringe pump under magnetic stirring. Upon completion, 7 mL of deionized water (pH 9.0) was added in one injection. After stirring for another 2 h, 1,4-dioxane was removed by dialysis against deionized water (pH 9.0; MWCO 3.5 kDa).

### Preparation of enzyme-loaded polymersomes

Typical procedures used for the encapsulation and removal of unloaded enzymes of enzymes including GOx, *α*-glucosidase, ALP, AcP, and Cat are described as follows.

Norbornene (Nor)-modified enzymes were synthesized at first (Supplementary Fig. 20a). Typical procedures employed for the synthesis of GOx-Nor are as follows. GOx (10 mg) was dissolved in PB buffer (20 mL, pH 7.4, 50 mM), and Nor-NHS (2 mg in 0.1 mL DMSO) was then added. The solution mixture was maintained at 5 °C for 48 h under magnetic stirring. Excess Nor-NHS and other impurities were removed by dialysis (MWCO 14 kDa) against deionized water. Subsequent lyophilization afforded GOx-Nor as a white powder. Other enzymes were modified with reactive norbornene moieties according to similar procedures. The activity of α-glucosidase, GOx, ALP, and AcP before and after modification was determined by monitoring the conversion of corresponding substances including *p*-nitrophenyl-*β*-D-glucopyranoside, oxygen, and *p*-nitrophenyl phosphate, respectively. The activity of *Nor*-modified enzymes (relative to intact unmodified enzymes) was calculated to be 0.91, 0.87, 0.92, and 0.80, respectively (Supplementary Fig. 21). As such, the current modification procedures provide a general and robust method to functionalize diverse enzymes.

Next, tetrazine-conjugated Sephadex G-20 dextran gel beads were prepared (Supplementary Fig. 20b). Sephadex G-20 dextran gel beads (1.0 g) were swollen by deionized water (50 mL) for 1 h. KIO_4_ (0.23 g, 1 mmol) was added and stirred for ~30 min. Upon addition of H_2_O_2_ (30%, 0.12 mL), the mixture was stirred at 60 °C for ~3 h. The resultant carboxyl-modified dextran gel beads were collected and washed with deionized water three times. Subsequently, carboxyl-modified dextran gel beads, Tz-NH_2_ (1.1 mmol), and NHS (1 mmol) were added into deionized water. After cooling to 4 °C, EDC (1.1 mmol) was slowly added, and the mixture was allowed to stir overnight at room temperature. Tetrazine-conjugated dextran gel beads (SG-20-Tz) were collected and thoroughly washed with water/EtOH (9/1, v/v) and deionized water three times, respectively.

Taking the encapsulation of GOx-Nor and Cat-Nor into **BPN**_**23**_ polymersomes as a typical example (Supplementary Fig. 20c), 2.0 mg **BPN**_**23**_ was dissolved in 1 mL 1,4-dioxane, and the solution was stirred and maintained at 25 °C in a water bath for 1 h. The aqueous solution of GOx-Nor (5.0 g/L) and Cat-Nor (1.0 g/L) in deionized water (1.0 mL, pH 9) was then added at a rate of 1.0 mL/h through a syringe pump under magnetic stirring (500 rpm). Upon completion, 8.0 mL deionized water (pH 9) was added, and the dispersion was stirred for another 5 h. SG-20-Tz dextran gel beads (100 mg) were then added, and the mixture was stirred at room temperature for 24 h. Upon removing the dextran gel beads, the remaining polymersome dispersion was further purified by brief dialysis (MWCO 14 kDa) against deionized water (pH 9.0) to remove 1,4-dioxane and other impurities. Notably, Nor-modified enzymes (GOx, Cat, α-glucosidase, ALP, and AcP) were used throughout this work for all enzyme-catalyzed experiments. To be concise, we used GOx instead of GOx-Nor and the same nomenclature was used for all other Nor-modified enzymes.

### Statistics and reproducibility

TEM, SEM, and CLSM experiments were repeated three times independently with similar results, and typical images are shown.

### Reporting summary

Further information on research design is available in the Nature Research Reporting Summary linked to this article.

## Supplementary information

Supplementary Information

Reporting Summary

## Data Availability

The data in this work are available in the manuscript or Supplementary Information, or available from the corresponding author upon request. Source data are provided with this paper.

## Source data

Source Data
