# Supplementary material for: Oscillating the local milieu of polymersome interiors via single input-regulated bilayer crosslinking and permeability tuning
Source: Nat Commun. 2022 Jan 31;13:585. doi: 10.1038/s41467-022-28227-6 (PMC8803951; doi:10.1038/s41467-022-28227-6)
Supplement: Supplementary file 1 — Supplementary Information [file 41467_2022_28227_MOESM1_ESM.pdf]

Supplementary Information

Oscillating the Local Milieu of Polymersome Interiors via Single Input-Regulated Bilayer Crosslinking and Permeability Tuning

Guhuan Liu, Jiajia Tan, Jie Cen, Guoying Zhang, Jinming Hu* and Shiyong Liu*

CAS Key Laboratory of Soft Matter Chemistry, Department of Polymer Science and Engineering, School of Chemistry and Materials Science, University of Science and Technology of China, Hefei, Anhui 230026, China.

Experimental Section

Materials. 4-Aminobenzyl alcohol (98%), 4-nitrobenzaldehyde, dibutyltin dilaurate (DBTL), doxorubicin hydrochloride (DOX·HCl), and Fmoc-Ala-Ala-OH dipeptide (Fmoc-AA-OH) were purchased from Sinopharm Chemical Reagent CO., Ltd. and used as received. 2,3,5,6-Tetrafluorobenzaldehyde, fluorescamine, 8-hydroxypyrene-1,3,6-trisulfonic acid trisodium salt (HPTS), tris(4,7-diphenyl-1,10-phenanthroline) ruthenium(II) dichloride, $[Ru(dpp)_3]Cl_2$, Nile red (NR), and *N*-(3-dimethylaminopropyl)-*N′*-ethylcarbodiimide hydrochloride (EDC) were purchased from J&K Scientific Ltd., and used as received. 2-Isocyanatoethyl methacrylate, di-*tert*-butyl chloromethyl phosphate, 2-dimethylaminoethanol, maltose, maltotriose, maltohexaose, glucose-6-phosphate (G6P), α-glucosidase, alkaline phosphatase (ALP), glucose oxidase (GOx), acid phosphatase (AcP), and catalase (Cat) were purchased from Sigma-Aldrich and used as received. SEPHADEX G-20 dextran gel was purchased from Sangon Biotechnology Co., Ltd. and used as received. Tetrahydrofuran (THF) was dried by refluxing over sodium shavings and distilled just prior to use. Fetal bovine serum (FBS), penicillin, streptomycin, and Dulbecco's modified Eagle's medium (DMEM) were purchased from GIBCO and used as received. 2,2-Azoisobutyronitrile (AIBN) was purified by recrystallization from 95% ethanol prior to use. Water was deionized with a Milli-Q SP reagent water system (Millipore) to a specific resistivity of 18.4 MΩ·cm. All other reagents were purchased from Sinopharm Chemical Reagent Co., Ltd. and used as received unless otherwise noted. Nile red-based methacrylate monomer (NRMA),[1] 7-nitro-1,2,3-benzoxadiazole-based methacrylamide monomer (NBDMA),[2] PEO-based macroRAFT agent (PEO_{45}-CTA),[3] 5-norbornene-2-carboxylic acid *N*-hydroxysuccinimide ester (Nor-NHS),[4] 4-(4,4,5,5-tetramethyl-1,3,2-dioxaborolan-2-yl)benzyl (4-methyl-2-oxo-2*H*-chromen-7-yl)carbamate (BAC),[5] (4-(6-methyl-1,2,4,5-tetrazin-3-yl)phenyl)methanamine (Tz-NH_2),[6] and poly(2-(dimethylamino)ethyl methacrylate) with a DP of ~45 ($PDMA_{45}$)[7] were synthesized according to previously reported literature procedures.

Synthesis of 2-((((4-((4-Nitrobenzylidene)amino)benzyl)oxy)carbonyl)amino)ethyl Methacrylate (NBI) Monomer. The synthesis of NBI monomer involves two steps as shown in Supplementary Fig. 1. Typical procedures used for the synthesis of 4-(4-nitrobenzylideneamino)benzyl alcohol (NBIA) are described below. 4-Aminobenzyl alcohol (1.23 g, 10 mmol) and 4-nitrobenzaldehyde (1.51 g, 10 mmol) were dissolved in anhydrous EtOH

(20 mL). The reaction mixture was stirred at 60 °C for 4 h; upon cooling down to 0 °C, the crude product was collected and further purified by recrystallization in EtOH three times, affording NBIA as a yellowish solid (~1.9 g, 74.2% yield). ^{1}H NMR (DMSO-d_6, 300 MHz, δ, ppm; Supplementary Fig. 2a): 8.56 (s, 1H, -N=C**H**-), 8.32 (d, 2H, aromatic proton), 8.09 (d, 2H, aromatic proton), 7.42 (d, 2H, aromatic proton), 7.26 (d, 2H, aromatic proton), and 4.74 (d, 2H, -C**H**$_2$-OH). ^{13}C NMR (DMSO-d6, 75 MHz, δ, ppm; Supplementary Fig. 2b): 157.20, 150.26, 149.48, 141.55, 139.92, 129.48, 127.97, 120.07, 121.17, and 64.70.

In the next step, NBIA (1.28 g, 5 mmol) was dissolved in THF (20 mL), a catalytic amount of DBTL (50 μL) and 2-isocyanatoethyl methacrylate (0.93 g, 6 mmol) were added. After stirring overnight at room temperature, all the solvents were removed on a rotary evaporator. The crude product was purified by recrystallization from *n*-hexane three times, affording NBI monomer as a yellowish solid (1.67 g, 81.1% yield). ^{1}H NMR ($CDCl_3$, 300 MHz, δ, ppm; Supplementary Fig. 3a): 8.57 (s, 1H, -N=C**H**-), 8.34 (d, 2H, aromatic proton), 8.08 (d, 2H, aromatic proton), 7.42 (d, 2H, aromatic proton), 7.25 (m, aromatic proton and $CDCl_3$), 6.13 (d, 1H, C**H**$_2$=C(CH_3)-), 5.59 (d, 1H, C**H**$_2$=C(CH_3)-), 5.11 (d, 2H, -C**H**$_2$-O-C(=O)-NH-), 4.21 (d, 2H, -NH-CH_2-C**H**$_2$-O-C(=O)-), 3.50 (d, 2H, -NH-C**H**$_2$-CH_2-O-C(=O)-), and 1.93 (s, 3H, CH_2=C(C**H**$_3$)-). ^{13}C NMR ($CDCl_3$, 75 MHz, δ, ppm; Supplementary Fig. 3b): 167.53, 157.99, 156.36, 150.68, 149.39, 141.32, 135.61, 129.77, 126.09, 124.05, 121.51, 66.85, 63.58, 40.79, and 18.78. ESI-MS (m/z, Supplementary Fig. 3c): $[M+H]^+$ calcd. for $C_{21}H_{22}N_3O_6$, 412.149; found, 412.149.

Synthesis of 2-((((4-((2,3,5,6-Tetrafluorobenzylidene)amino)benzyl)oxy)carbonyl) amino)ethyl methacrylate *(TFI) Monomer.* As shown in Supplementary Fig. 1, TFI monomer synthesis also involves two steps. For the synthesis of 4-(2,3,5,6-tetrafluorobenzylideneamino)benzyl alcohol (TFIA), 2,3,5,6-tetrafluorobenzaldehyde (5.34 g, 30 mmol) and 5 μL acetic acid (90 μmol) were added into the stirred suspension of 4-aminobenzyl alcohol (1.23 g, 10 mmol) in 50 mL benzene. The reaction mixture was heated to reflux for ~17 h and water generated during the reaction was removed using a Dean-Stark apparatus. After removing all the solvents under reduced pressure, the crude product was further purified by recrystallization in *n*-hexane three times, affording TFIA as a yellowish solid (2.23 g, 78.8% yield). ^{1}H NMR (DMSO-d_6, 300 MHz, δ, ppm; Supplementary Fig. 4a): 8.69 (s, 1H, -N=C**H**-), 8.08 (d, 1H, aromatic proton), 7.40 (d, 2H, aromatic proton), 7.32 (d, 2H, aromatic

proton), 5.26 (m, 1H, -CH_2-O**H**) and 4.53 (d, 2H, -C**H**$_2$-OH). ^{13}C NMR (DMSO-d_6, 75 MHz, δ, ppm; Supplementary Fig. 4b): 149.68, 149.23, 144.03, 141.93, 127.24, 120.82, 116.10, 108.14, and 62.14.

In the next step, TFIA (1.98 g, 7 mmol) was dissolved in THF (20 mL), followed by the addition of a catalytic amount of DBTL (50 μL) and 2-isocyanatoethyl methacrylate (1.55 g, 10 mmol). After stirring overnight at room temperature, all the solvents were removed on a rotary evaporator. The crude product was purified by recrystallization in *n*-hexane three times, affording TFI monomer as a yellowish solid (2.33 g, 75.8%). ^{1}H NMR (DMSO-d_6, 300 MHz, δ, ppm; Supplementary Fig. 5a): 8.68 (s, 1H, -N=C**H**-), 8.13 (m, 1H, aromatic proton), 7.4~7.2 (m, 4H, aromatic proton), 6.04 (d, 1H, C**H**$_2$=C(CH_3)-), 5.65 (d, 1H, C**H**$_2$=C(CH_3)-), 5.04 (d, 2H, -C**H**$_2$-O-C(=O)-NH-), 4.12 (d, 2H, -NH-CH_2-C**H**$_2$-O-C(=O)-), 3.33 (d, 2H, -NH-C**H**$_2$-CH_2-O-C(=O)-), and 1.86 (s, 3H, CH_2=C(C**H**$_3$)-). ^{13}C NMR (DMSO-d_6, 75 MHz, δ, ppm; Supplementary Fig. 5b): 166.48, 156.47, 150.23, 147.04, 144.20, 135.74, 129.92, 125.72, 120.82, 115.91, 108.93, 65.19, 63.09, and 17.65. ESI-MS (m/z, Supplementary Fig. 5c): $[M+H]^+$ calcd. for $C_{21}H_{19}F_4N_2O_4$, 439.126; found, 439.126.

Synthesis of Zwitterionic PMN (Fig. S7). Di-*tert*-butyl chloromethyl phosphate and 2-dimethylaminoethanol were dissolved in MeCN at first. After stirring at 50 °C overnight and cooling down to room temperature, the crude product was collected by centrifugation and washed with cold THF three times. The resultant product was dissolved in DCM, and TFA was added. The mixture was stirred at room temperature for 4 h. After removing all the solvents, the residues were collected and thoroughly washed with cold THF three times, affording zwitterionic PMN as a white solid. ^{1}H NMR (D_2O, 300 MHz, δ, ppm, Supplementary Fig. 32a): 4.83 (d, 2H, -O-C**H**$_2$-N^+(CH_3)$_2$-), 3.95 (m, 2H, HO-CH_2-C**H**$_2$-N^+(CH_3)$_2$-), 3.43 (t, 2H, HO-C**H**$_2$-CH_2-N^+(CH_3)$_2$-), 3.06 (s, 6H, HO-CH_2-CH_2-N^+(C**H**$_3$)$_2$-). ^{13}C NMR (D_2O, δ, ppm, Supplementary Fig. 32b): 84.8, 62.2, 55.1, 48.3. ^{31}P NMR (D_2O, δ, ppm, Supplementary Fig. 32c): -2.5. ESI-MS (m/z, Supplementary Fig. 32d): $[M + Na]^+$ calcd. for $C_5H_{14}NO_4PNa$, 222.050; found, 222.050.

*Synthesis of **BPN_n** and **BPF_n** Diblock Copolymers.* Typical procedures employed for the RAFT synthesis of **BPN_{23}** are as follows. NBI monomer (1.00 g, 2.43 mmol), PEO_{45}-CTA (0.18 g, 0.081 mmol), and AIBN (1.3 mg, 8.1 μmol) were dissolved in DMF (1.0 mL) in a reaction tube equipped with a magnetic stirring bar. The tube was carefully degassed by three freeze-

pump-thaw cycles and then sealed under vacuum. After being thermostated at 70 °C in an oil bath and stirred for 6 h, the reaction tube was quenched into liquid nitrogen and opened; the mixture was then precipitated into an excess of diethyl ether. The above dissolution-precipitation cycle was repeated three times. PEO_{45}-*b*-$PNBI_n$ diblock copolymer, **BPN**, was obtained as a yellowish powder (1.28 g, 81.2% yield). GPC analysis revealed an M_n of 33.9 kDa and an M_w/M_n of 1.18 (Supplementary Fig. 6). The degree of polymerization, DP, of PNBI block was determined to be ~23 by ^{1}H NMR analysis, thus the diblock copolymer was denoted as **BPN_{23}**. According to similar procedures, **BPN_n** (n = 8, 36, and 40) and **BPF_n** (n = 6, 25, and 35) diblock copolymers, together with NBD-labeled and Nile red-labeled diblock copolymers, **BPN_{21}-*NBD***, **BPN_{24}-*NR***, **BPF_{25}-*NBD***, and **BPF_{25}-*NR*** were also synthesized. Structural parameters of all pH-reactive diblock copolymers synthesized in this work are summarized in Table 1.

Fabrication of Polymersomes Encapsulating DOX·HCl, Fluorescent Probes, and Self-Assembling Dipeptide. Typical procedures for the encapsulation of the water-soluble drug of DOX·HCl, probe molecules of HPTS and $[Ru(dpp)_3]Cl_2$, and dipeptide of Fmoc-AA-OH into polymersomes are described as follows. 2.0 mg diblock copolymer was dissolved in 1 mL 1,4-dioxane, and the solution was stirred and maintained at 25 °C in a water bath for 1 h. Next, 2 mg DOX·HCl was dissolved in 2.0 mL deionized water, and the aqueous solution was slowly added within 1 h into the above copolymer solution in 1,4-dioxane, followed by the injection of 7 mL deionized water (pH 9.0). After stirring for another 2 h, 1,4-dioxane and free drug were briefly removed by dialysis (MWCO 3.5 kDa) against deionized water (pH 9.0). Based on the standard calibration curve, the DOX loading content was calculated to be ~5.2%. According to similar procedures, water-soluble fluorescent probes and dipeptide included HPTS, $[Ru(dpp)_3]Cl_2$, and Fmoc-AA-OH were also encapsulated into polymersome interiors.

In Vitro Drug Release Measurements. Typically, 150 μL DOX-loaded **BPN_{23}** polymersome dispersion (0.2 g/L) was placed in a dialysis tube (cellulose membrane; MWCO 3.5 kDa) and then immersed into 5 mL buffer medium (10 mM, 37 °C) with pH set at 5.0, 5.5, 6.0, and 7.4. At specified time intervals, the external buffer medium was removed and replaced with fresh buffer medium. Upon each replacement, 1.0 mL external buffer medium was taken and mixed with 9.0 mL DMSO. The DOX concentration was quantified by fluorescence measurements against the standard calibration curve.

In Vitro Cytotoxicity Assay. Cell viabilities were determined by the MTT assay. HepG2 cells were seeded in a 96-well plate at an initial density of ca. 7000 cells/well in 100 μL complete DMEM medium. After incubating for 24 h, the culture was replaced with fresh DMEM medium, and the cells were treated with blank and DOX-loaded polymersome dispersions at varying concentrations. Cells were then incubated with 5% CO_2 at 37 ^{o}C for 48 h. The culture medium in each well was removed and replaced with 100 μL of DMSO. The plate was gently agitated for 15 min before the absorbance at 570 nm was recorded on a microplate reader (Thermo Fisher Scientific).

Characterization. All nuclear magnetic resonance (NMR) spectra were recorded on a Bruker AV300 NMR 300 MHz spectrometer operated in the Fourier transform mode. $CDCl_3$, DMSO-d_6, and D_2O were used as solvents. Molecular weights and molecular weight distributions were determined by gel permeation chromatography (GPC) equipped with Agilent G1310B pump and Wyatt Optilab refractive index detector (λ = 658 nm) (set at 50 ^{o}C). It used a series of two linear Shodex MZ Columns at an oven temperature of 50 ^{o}C. The eluent was DMF (containing 10 mM LiBr) at a flow rate of 1.0 mL/min. A series of low polydispersity polystyrene standards were employed for calibration. Dynamic laser light scattering (DLS) measurements were conducted on a commercial spectrometer (ALV/DLS/SLS-5022F) equipped with a multi-tau digital time correlator (ALV5000) and a cylindrical 22 mW UNIPHASE He-Ne laser (λ_0= 632 nm) as the light source. Scattered light was collected at a fixed angle of 90° for a duration of ~5 min. Distribution averages and particle size distributions were computed using cumulants analysis and CONTIN routines. All data were averaged over three measurements. Transmission electron microscopy (TEM) observations were conducted on a Hitachi H-800 electron microscope at an acceleration voltage of 200 kV. Samples for TEM observation were prepared by placing 10 μL of polymersome dispersions (0.5 g/L) on copper grids successively coated with thin films of Formvar and carbon. No staining was applied for TEM observations. Cryo-TEM observations were conducted on an FEI Talos F200C microscope operating at 200 kV. Specifically, an aqueous dispersion (10 μL) of **BPN$_{23}$** was dropped onto a lacey carbon-film-supported TEM copper grid. All TEM grids used for cryo-TEM imaging were pretreated with plasma air to render the lacey carbon film hydrophilic. Confocal laser scanning microscopy (CLSM) images were acquired using a Leica TCS SP5 microscope. UV-Vis absorption spectra

were collected on a UV-3600 Plus spectrophotometer (Shimadzu). Fluorescence spectra were recorded on an F-4600 (Hitachi) spectrofluorometer. The slit widths were both set at 5 nm for excitation and emission.

References

1. Yao C, Li Y, Wang Z, Song C, Hu X, Liu S. Cytosolic NQO1 Enzyme-Activated Near-Infrared Fluorescence Imaging and Photodynamic Therapy with Polymeric Vesicles. *ACS Nano* **14**, 1919-1935 (2020).
2. Yin J, Hu HB, Wu YH, Liu SY. Thermo-and light-regulated fluorescence resonance energy transfer processes within dually responsive microgels. *Polym. Chem.* **2**, 363-371 (2011).
3. Wang XR, Liu GH, Hu JM, Zhang GY, Liu SY. Concurrent Block Copolymer Polymersome Stabilization and Bilayer Permeabilization by Stimuli-Regulated "Traceless" Crosslinking. *Angew. Chem. Int. Ed.* **53**, 3138-3142 (2014).
4. Carrillo A, Yanjarappa MJ, Gujraty KV, Kane RS. Biofunctionalized block copolymer nanoparticles based on ring-opening metathesis polymerization. *J. Polym. Sci. Part A: Polym. Chem.* **44**, 928-939 (2006).
5. Deng ZY*, et al.* Engineering Intracellular Delivery Nanocarriers and Nanoreactors from Oxidation-Responsive Polymersomes via Synchronized Bilayer Cross-Linking and Permeabilizing Inside Live Cells. *J. Am. Chem. Soc.* **138**, 10452-10466 (2016).
6. Zuo L*, et al.* Coordinating bioorthogonal reactions with two tumor-microenvironment-responsive nanovehicles for spatiotemporally controlled prodrug activation. *Chem. Sci.* **11**, 2155-2160 (2020).
7. Liu SY, Weaver JVM, Tang YQ, Billingham NC, Armes SP, Tribe K. Synthesis of shell cross-linked micelles with pH-responsive cores using ABC triblock copolymers. *Macromolecules* **35**, 6121-6131 (2002).

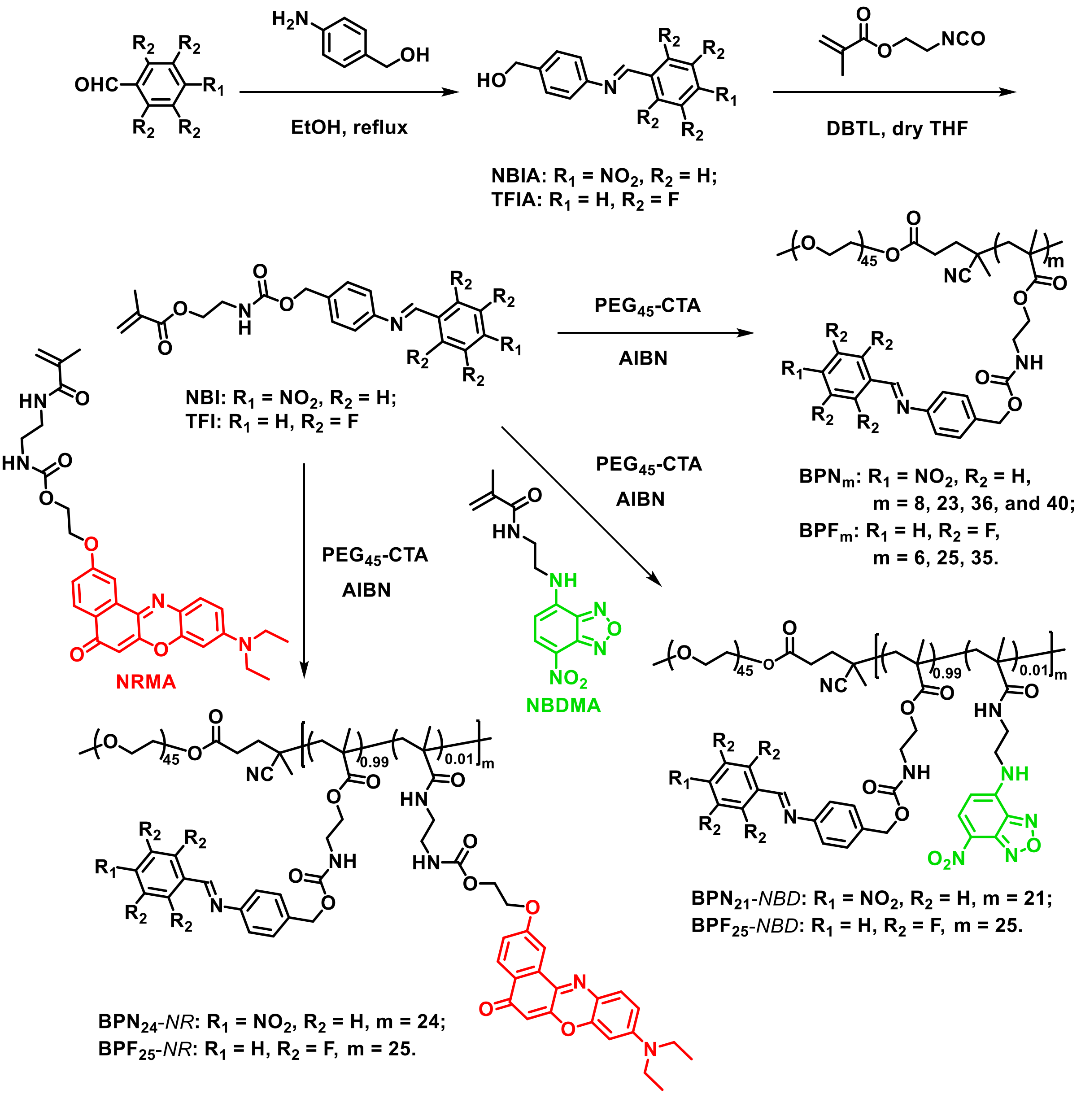

Supplementary Fig. 1 Synthetic routes employed for the synthesis of pH-responsive **NBI** and **TFI** monomers, amphiphilic **BPN**$_{\mathbf{m}}$ and **BPF**$_{\mathbf{m}}$ diblock copolymers, and dye-labeled **BPN**$_{\mathbf{21}}$**-***NBD*, **BPF**$_{\mathbf{25}}$**-***NBD*, **BPN**$_{\mathbf{21}}$**-***NR*, and **BPF**$_{\mathbf{25}}$**-***NR* copolymers via reversible addition-fragmentation chain transfer (RAFT) polymerizations using PEO-based macroRAFT agent.

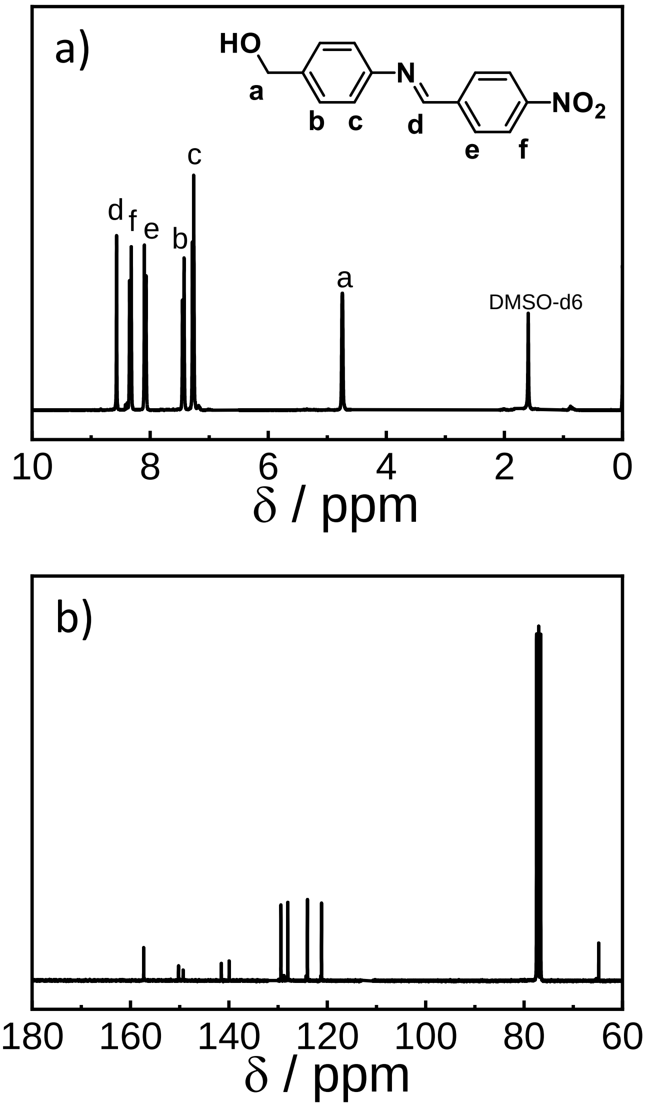

Supplementary Fig. 2 (a) ^{1}H NMR and (b) ^{13}C NMR spectra recorded for 4-(benzylideneamino)benzyl alcohol (NBIA) in DMSO-d_6.

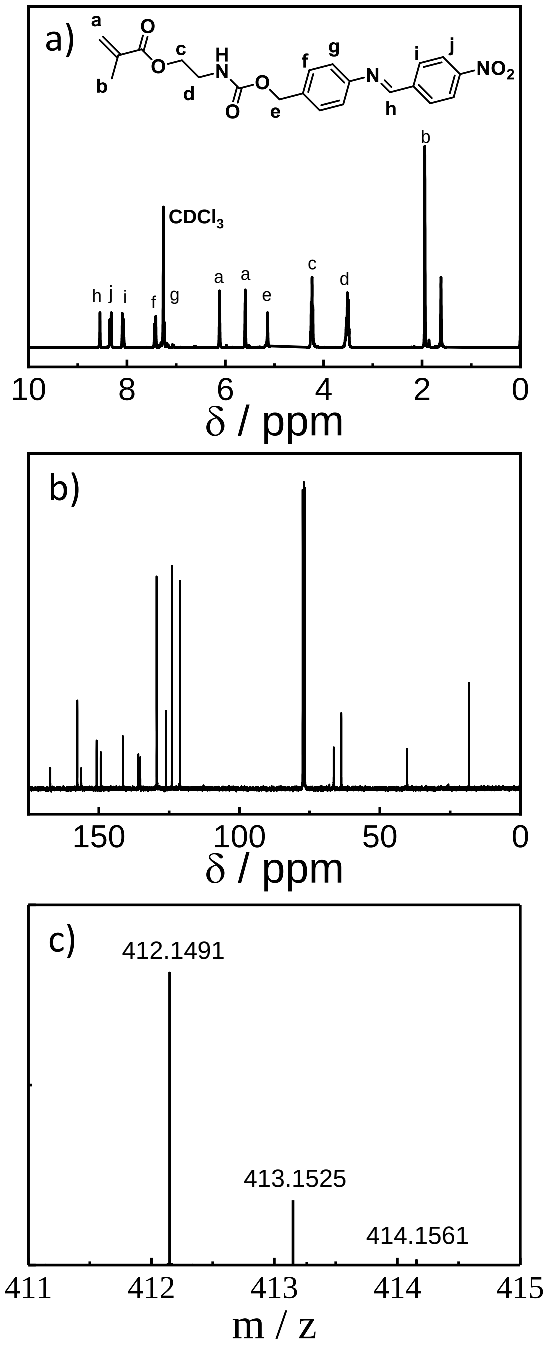

Supplementary Fig. 3 (a) ^{1}H and (b) ^{13}C NMR spectra recorded for NBI monomer in $CDCl_3$. (c) ESI mass spectrum recorded for NBI monomer.

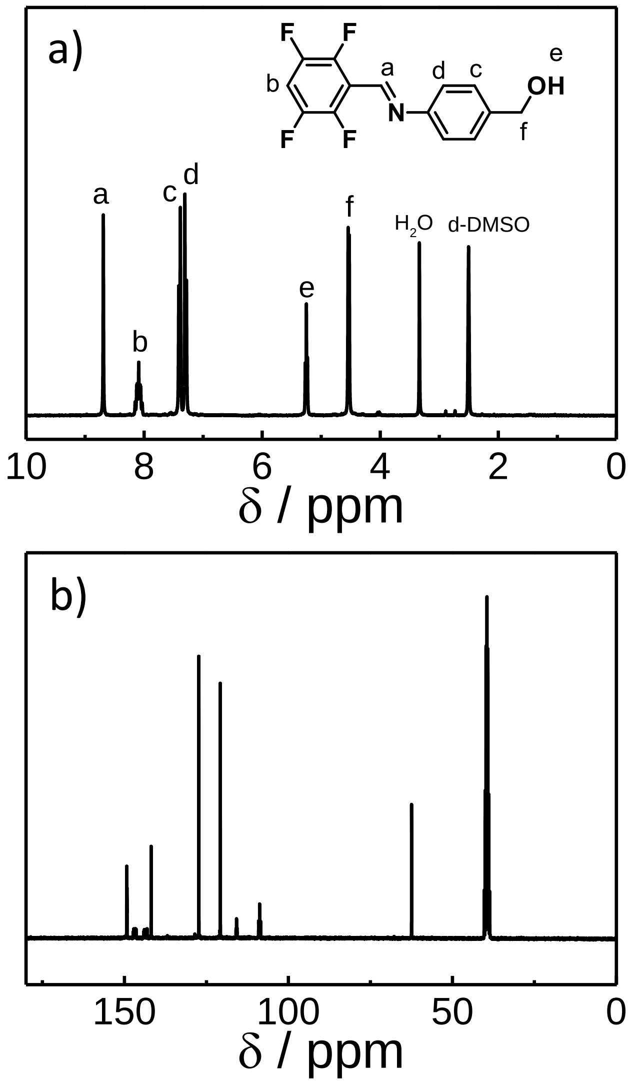

Supplementary Fig. 4 (a) ^{1}H and (b) ^{13}C NMR spectra recorded for 4-(2,3,5,6-tetrafluorobenzylideneamino)benzyl alcohol (TFIA) in DMSO-d_6.

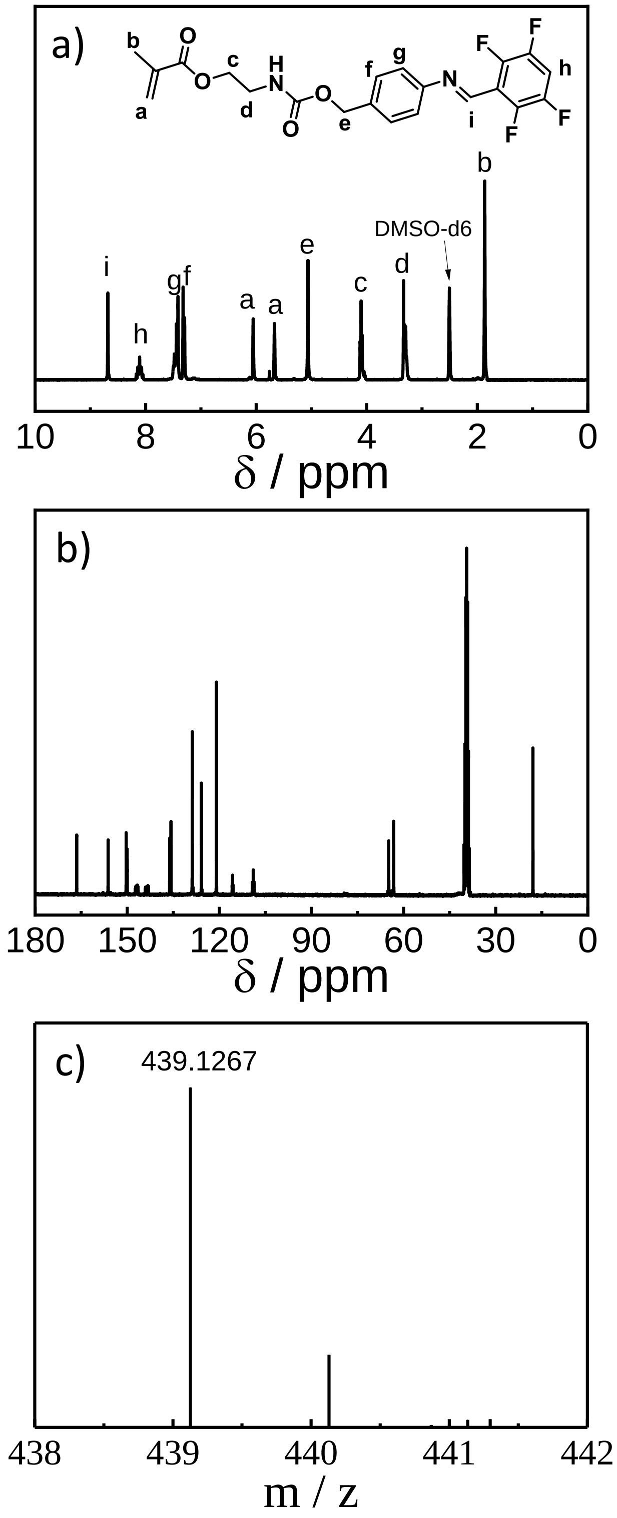

Supplementary Fig. 5 (a) ^{1}H and (b) ^{13}C NMR spectra recorded for TFI monomer in DMSO-d_6. (c) ESI mass spectrum recorded for TFI monomer.

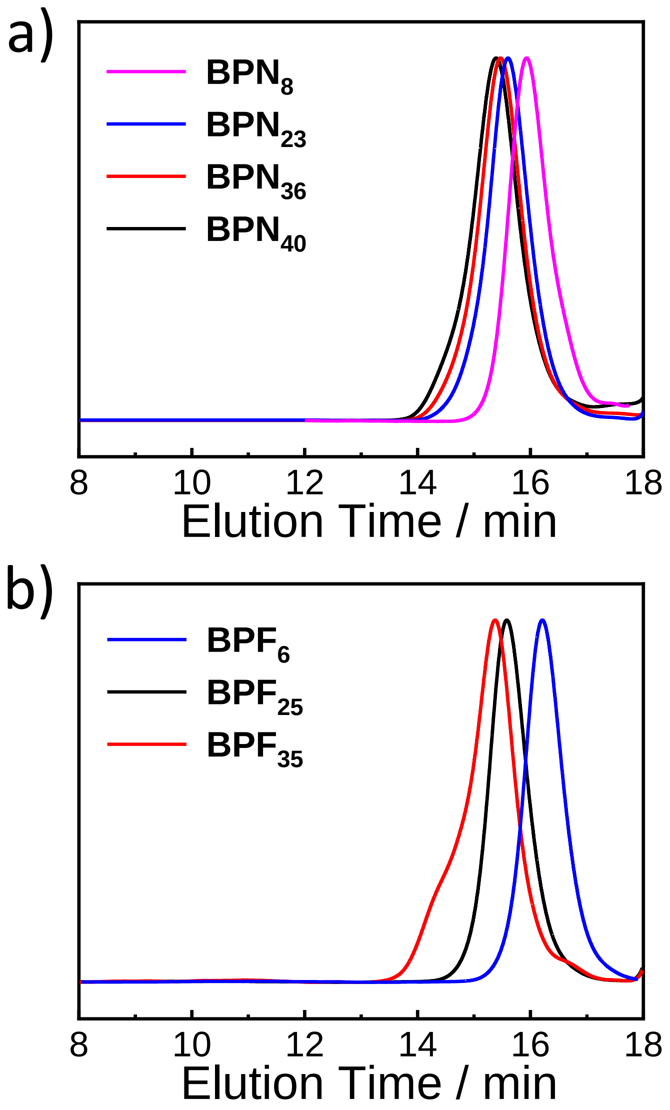

Supplementary Fig. 6 GPC traces recorded for (a) **BPN$_n$** and (b) **BPF$_n$** amphiphilic diblock copolymers using DMF as eluent.

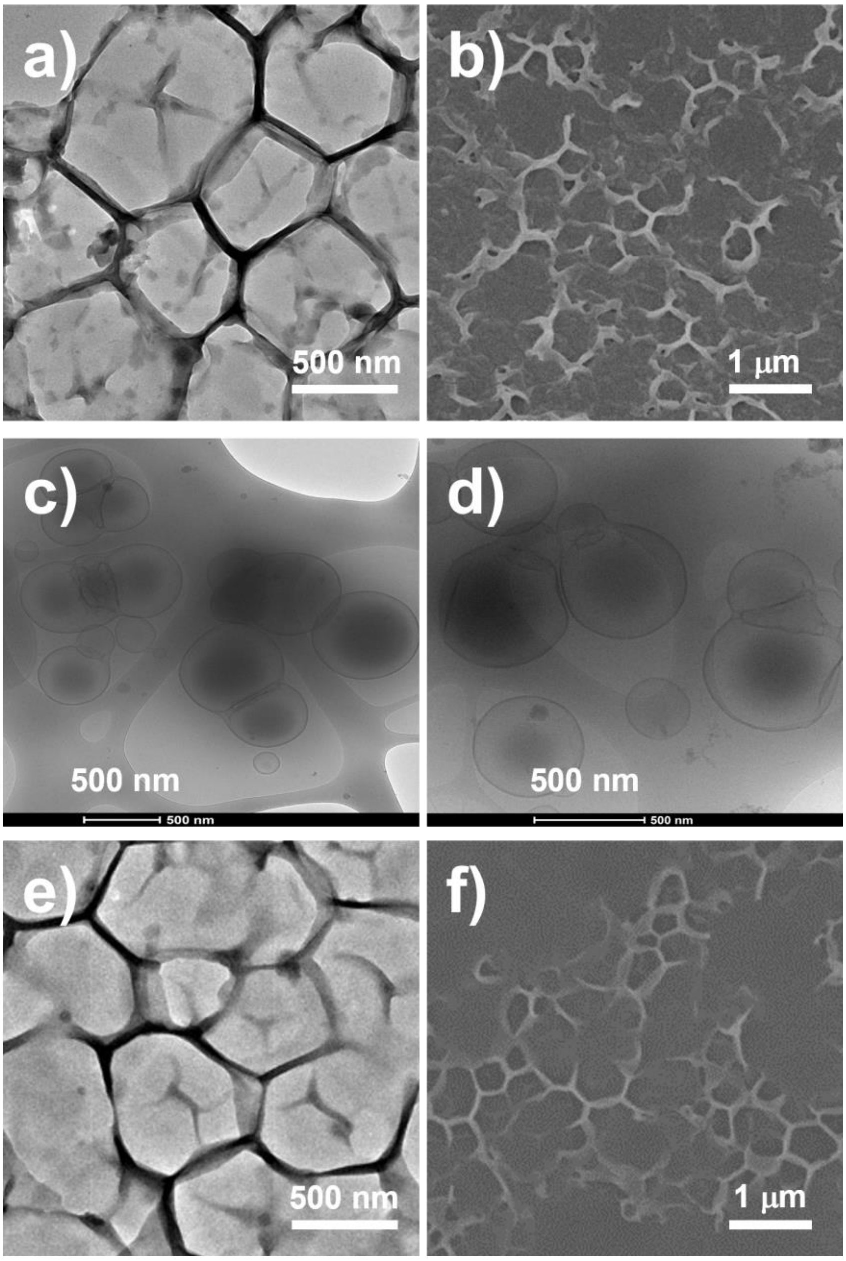

Supplementary Fig. 7 Typical TEM (a and b), cryo-TEM (c and d), and SEM (e and f) images recorded for **BPN$_{23}$** (a-d) and **BPF$_{25}$** (e and f) vesicles.

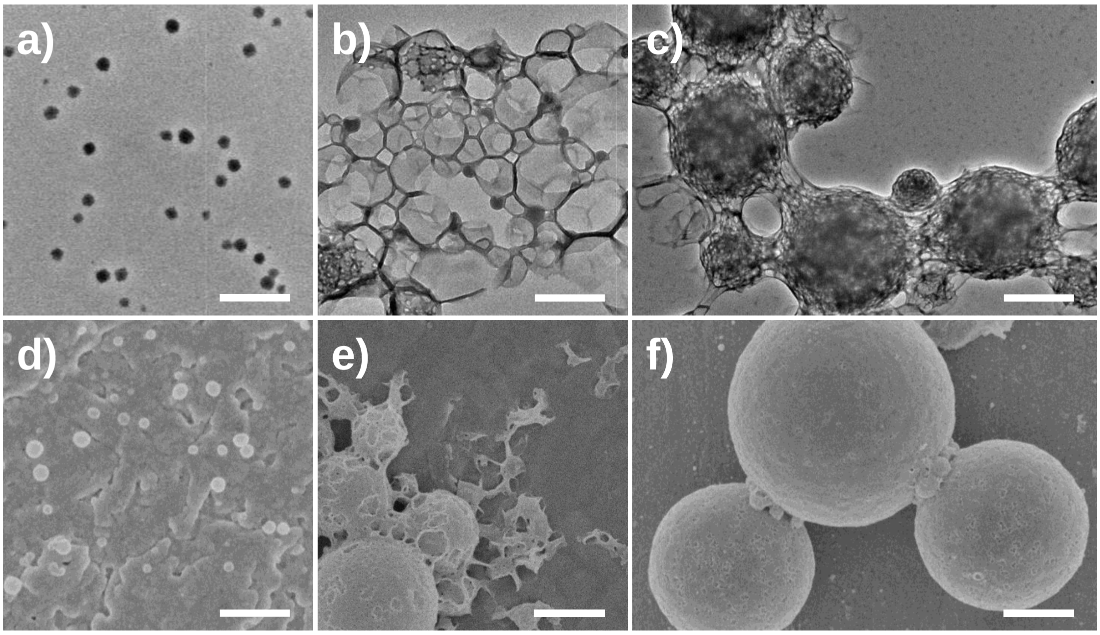

Supplementary Fig. 8 Typical TEM (a-c) and SEM (d-f) images (scale bars: 500 nm) recorded for nanoassemblies of **BPN_8** (a and d), **BPN_{36}** (b and e), and **BPN_{40}** (c and f).

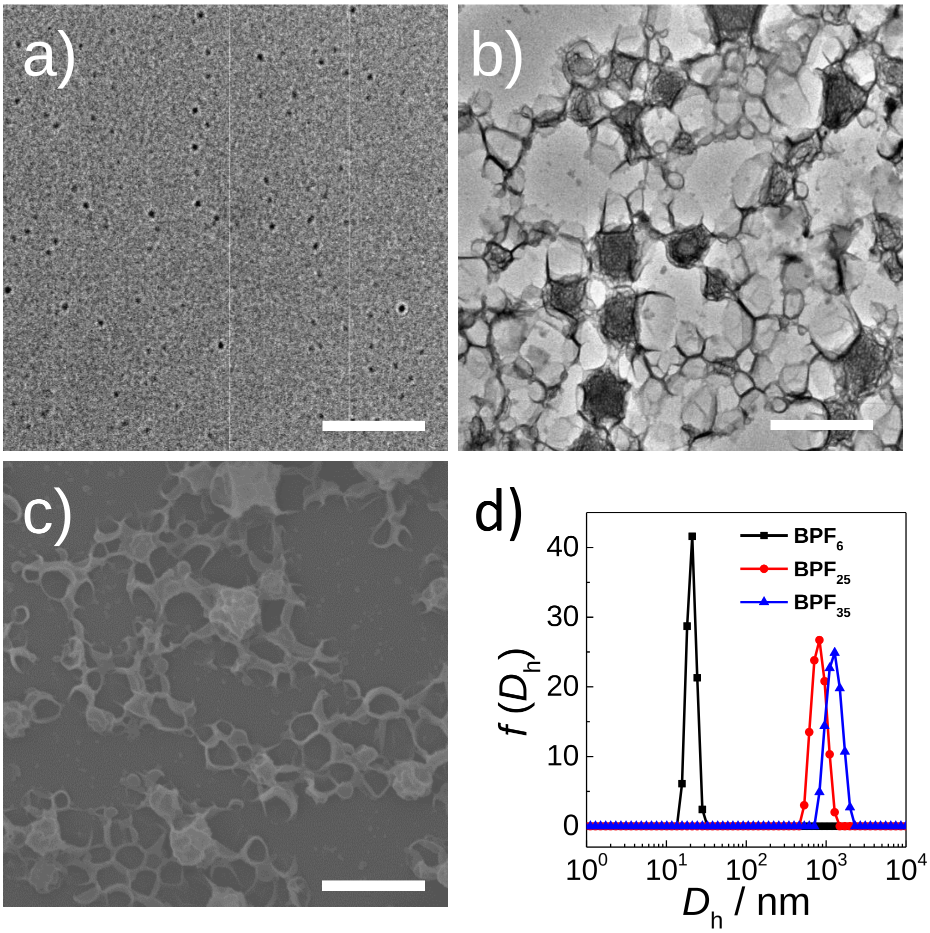

Supplementary Fig. 9 Typical TEM (a and b) and SEM (c) images (scale bars: 1 μm) recorded for nanoassemblies of **BPF$_6$** (a) and **BPF$_{35}$** (b and c). (d) Intensity-average hydrodynamic distributions, $f(D_h)$, recorded for aqueous dispersions (0.2 g/L) of **BPF$_6$**, **BPF$_{25}$**, and **BPF$_{35}$** nanoassemblies.

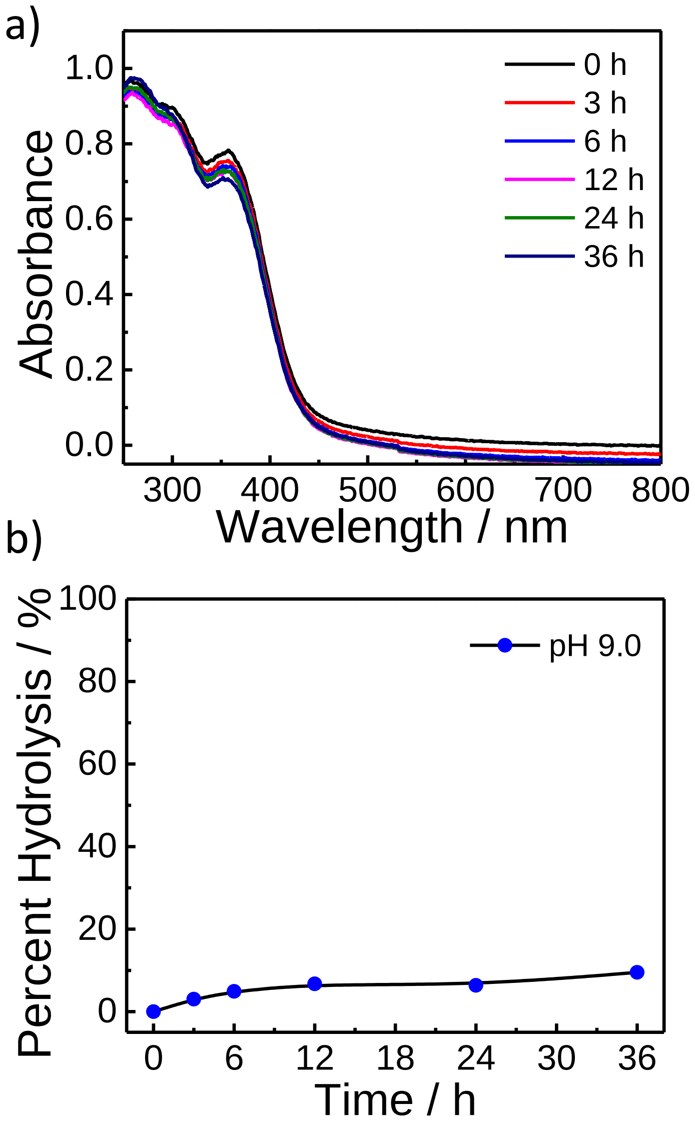

Supplementary Fig. 10 (a) Time-dependent evolution of UV-Vis absorption spectra recorded for **BPN23** polymersome dispersion (0.2 g/L) at pH 9.0. (b) Hydrolysis kinetics recorded for **BPN23** polymersome dispersion at pH 9.0.

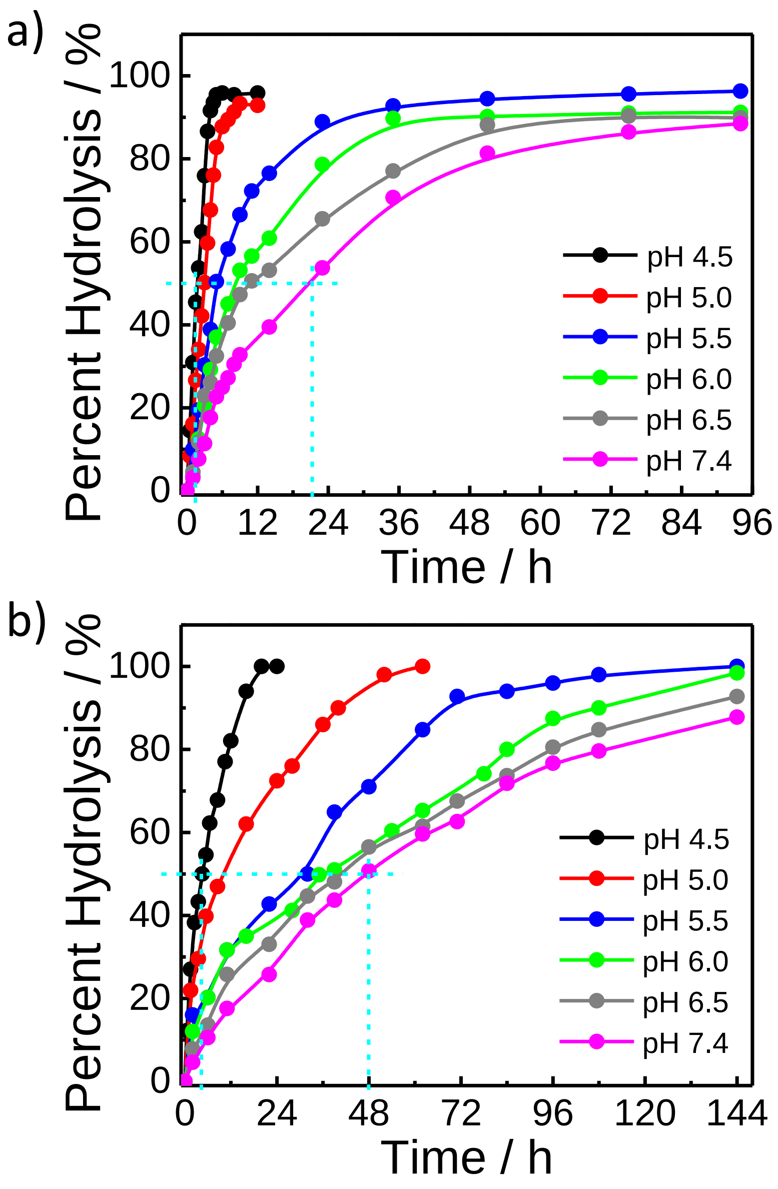

Supplementary Fig. 11 Hydrolysis kinetics recorded for **BPN$_{23}$** (a) and **BPF$_{25}$** (b) polymersome dispersions (0.2 g/L, ~400 µM imine linkages) at varying pH conditions, as monitored by time-dependent changes in UV-Vis absorbance intensities at wavelengths of 355 and 335 nm, respectively (see Fig. 2a for details).

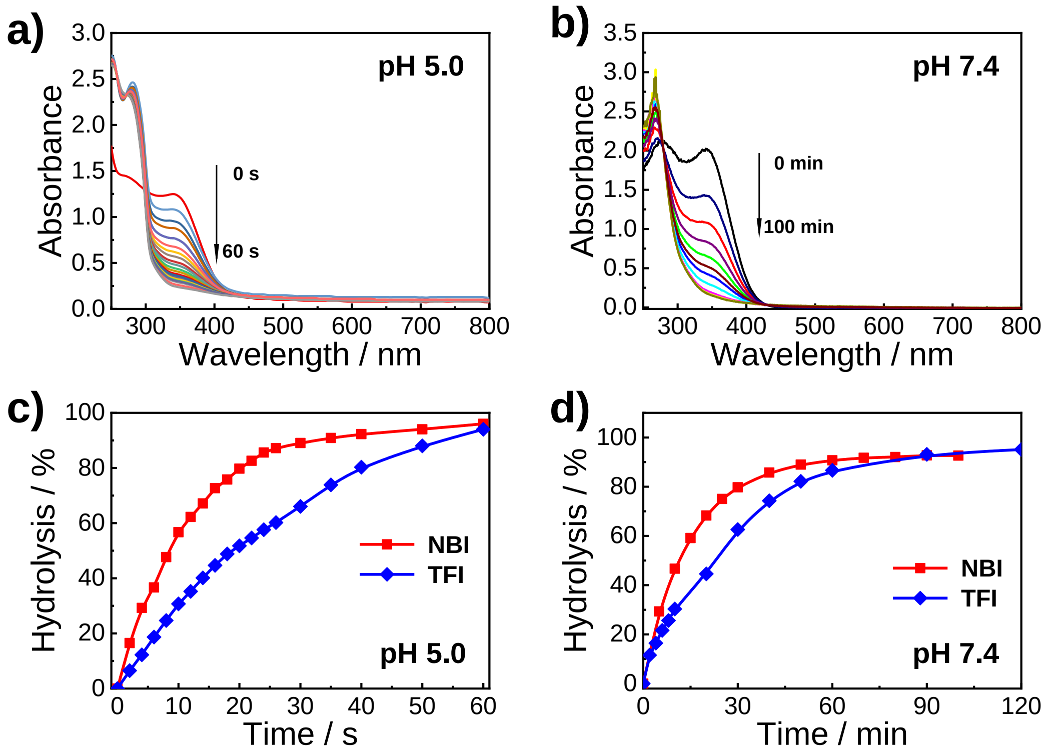

Supplementary Fig. 12 (a and b) Time-dependent evolution of UV-Vis absorption spectra of NBI and corresponding (c and d) hydrolysis kinetics recorded for 0.4 mM **NBI** and **TFI** solution in DMSO/water mixture (3/7 v/v) at pH 5.0 (a and c) and 7.4 (b and d), respectively.

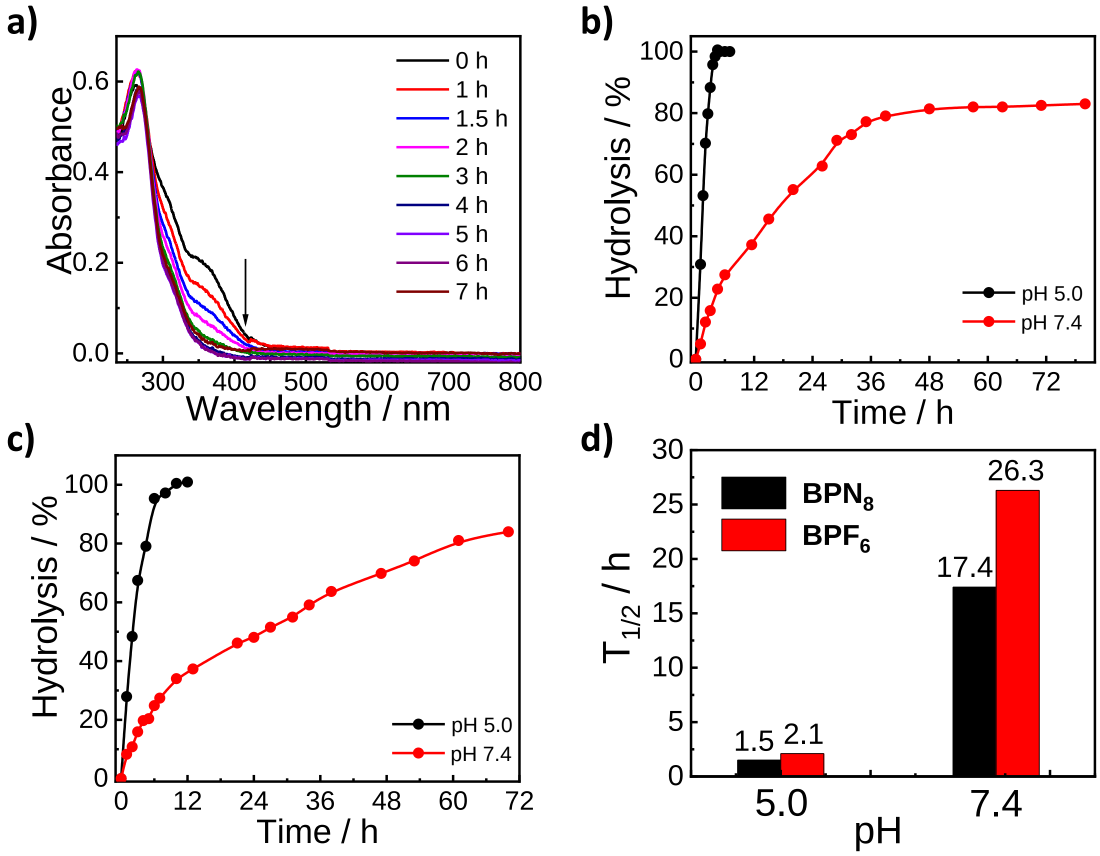

Supplementary Fig. 13 (a) Time-dependent evolution of UV-Vis absorption spectra recorded for **BPN8** micellar dispersion (0.26 g/L, ~400 μM imine linkages) at pH 5.0. Hydrolysis kinetics recorded for **BPN8** (b) and **BPF6** (c) micellar dispersions (0.26 g/L, ~400 μM imine linkages) at pH 5.0 and pH 7.4, respectively. (d) Summary of hydrolysis half-lives of **BPN8** and **BPF6** micellar dispersions at pH 5.0 and pH 7.4, respectively.

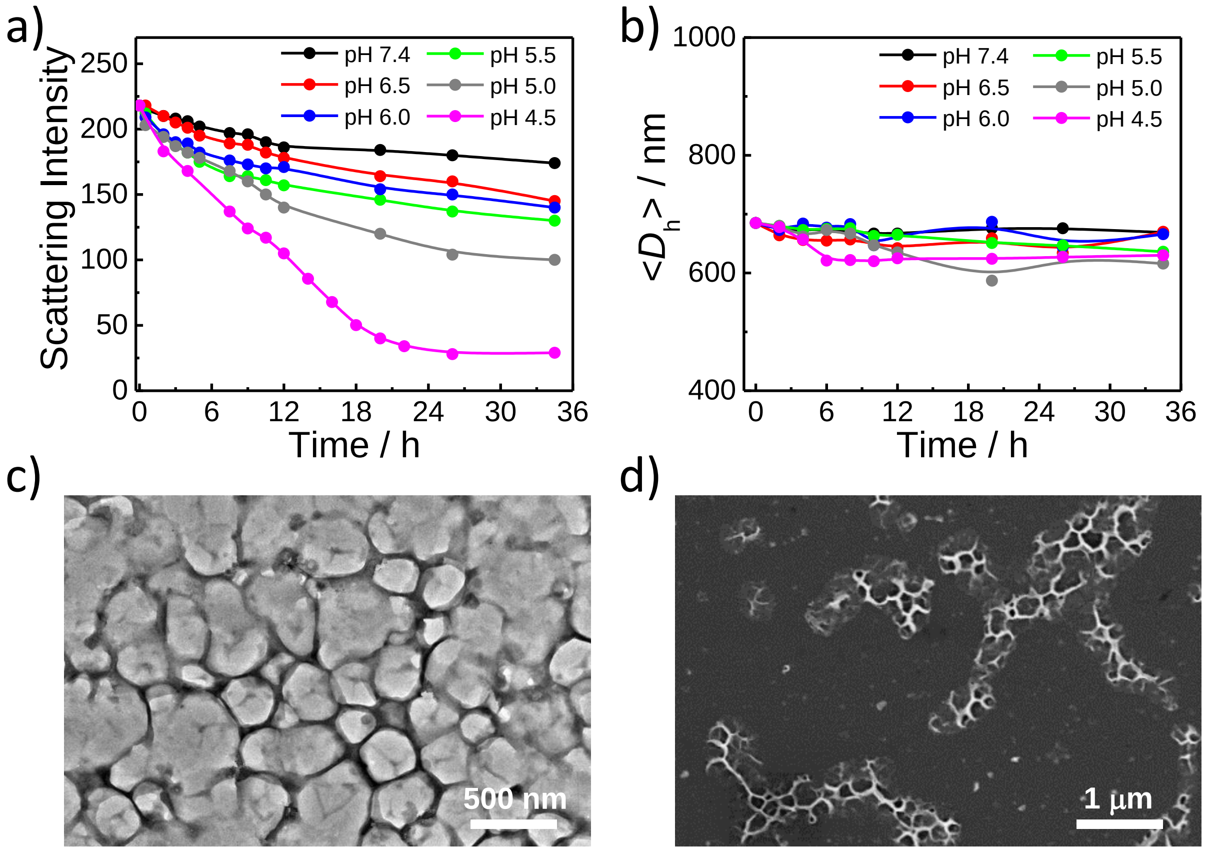

Supplementary Fig. 14 (a) Time-dependent evolution of scattered light intensities and (b) intensity-average hydrodynamic diameters, <D_h>, recorded for **BPF25** polymersome dispersion upon incubating at varying pH conditions. Typical TEM (c) and SEM (d) images of **BPF25** polymersomes after hydrolysis at pH 5.0 for 72 h.

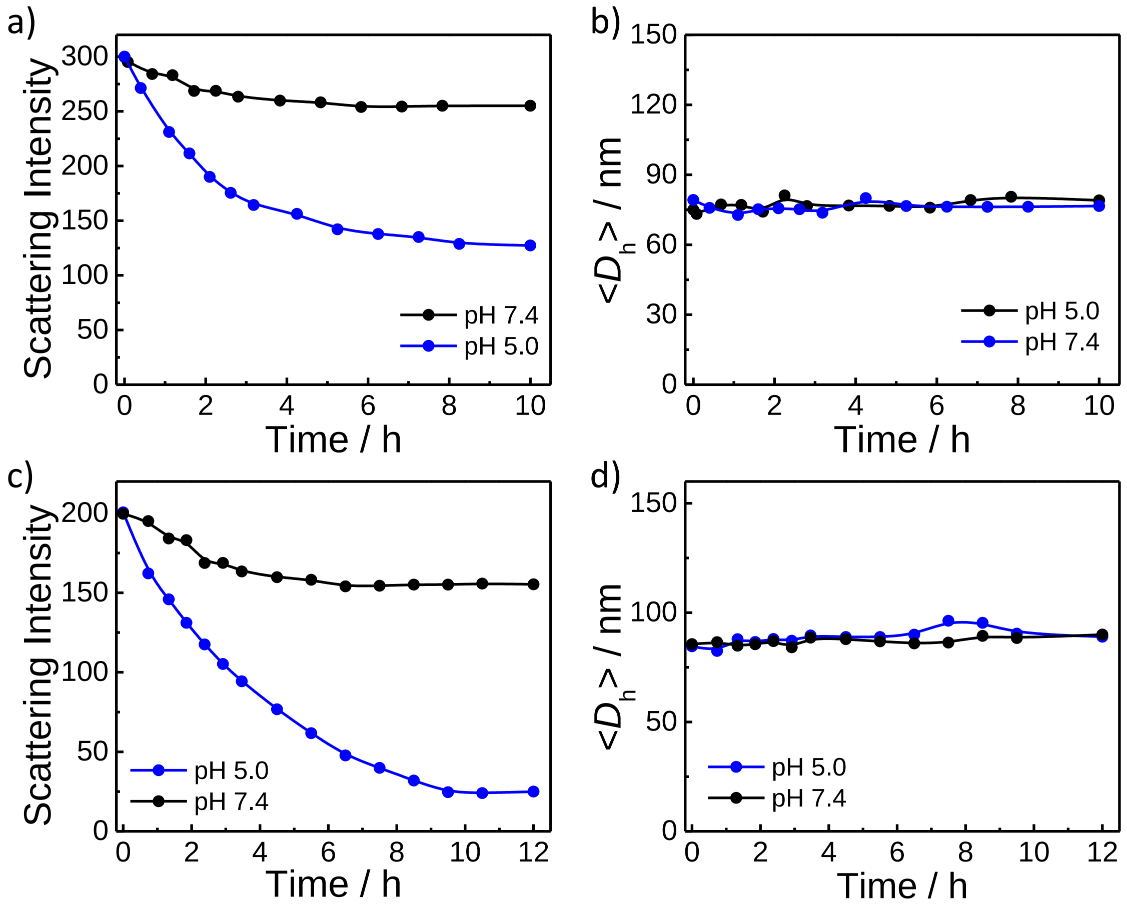

Supplementary Fig. 15 (a and c) Time-dependent evolution of scattered light intensities and (b and d) intensity-average hydrodynamic diameters, <D_h>, recorded for **BPN$_8$** (a and b) and **BPF$_6$** (c and d) micellar dispersions (0.26 g/L) upon incubating at pH 5.0 and pH 7.4, respectively.

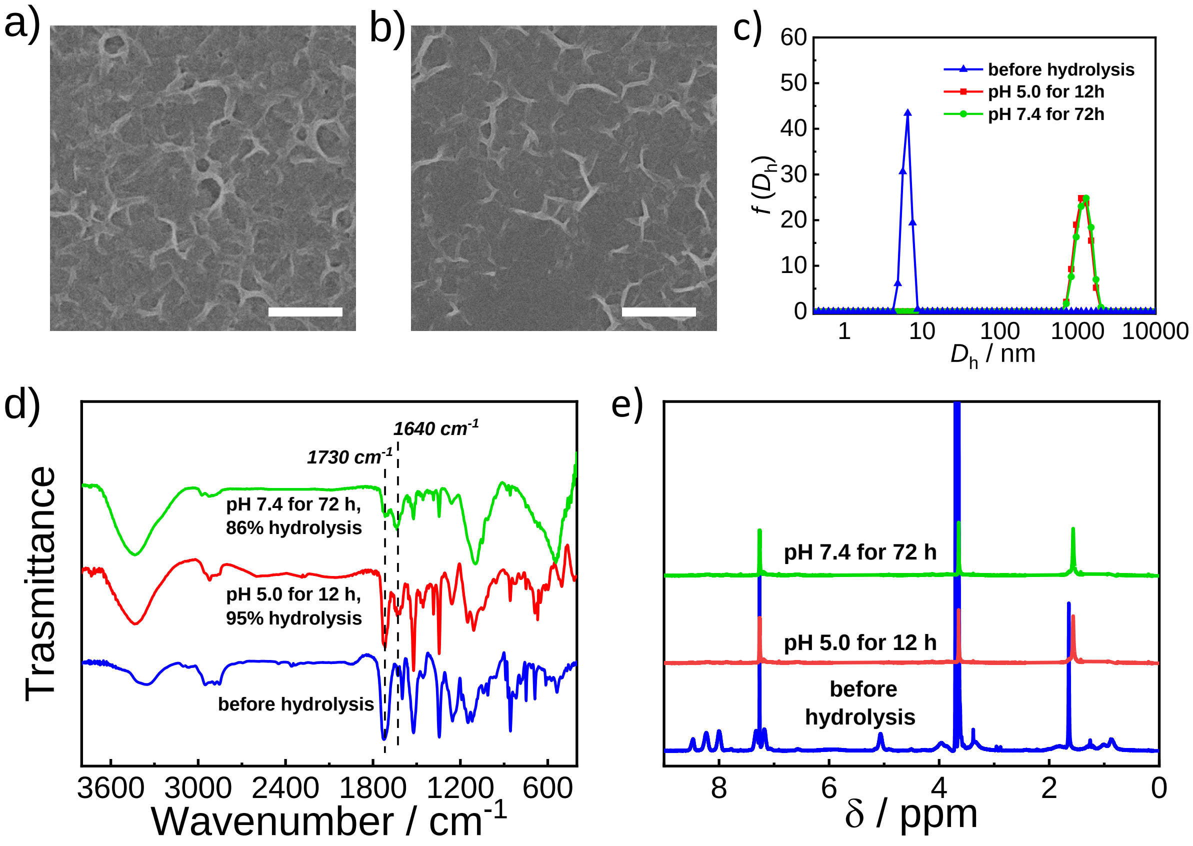

Supplementary Fig. 16 SEM images recorded for **BPN**$_{\mathbf{23}}$ polymersomes after being hydrolyzed at (a) pH 5.0 for 12 h and (b) pH 7.4 for 72 h. (c) Intensity-average hydrodynamic distributions, $f(D_h)$, recorded for **BPN**$_{\mathbf{23}}$ polymersome dispersions before and after hydrolysis at pH 5.0 for 12 h and at pH 7.4 for 72 h, followed by 10-fold dilution with DMSO. (d) FT-IR spectra and (e) ^{1}H NMR spectra (in $CDCl_3$) recorded for **BPN**$_{\mathbf{23}}$ polymersomes before and after hydrolysis; samples were obtained by lyophilizing polymersome dispersions before and after hydrolysis at pH 5.0 for 12 h and at pH 7.4 for 72 h.

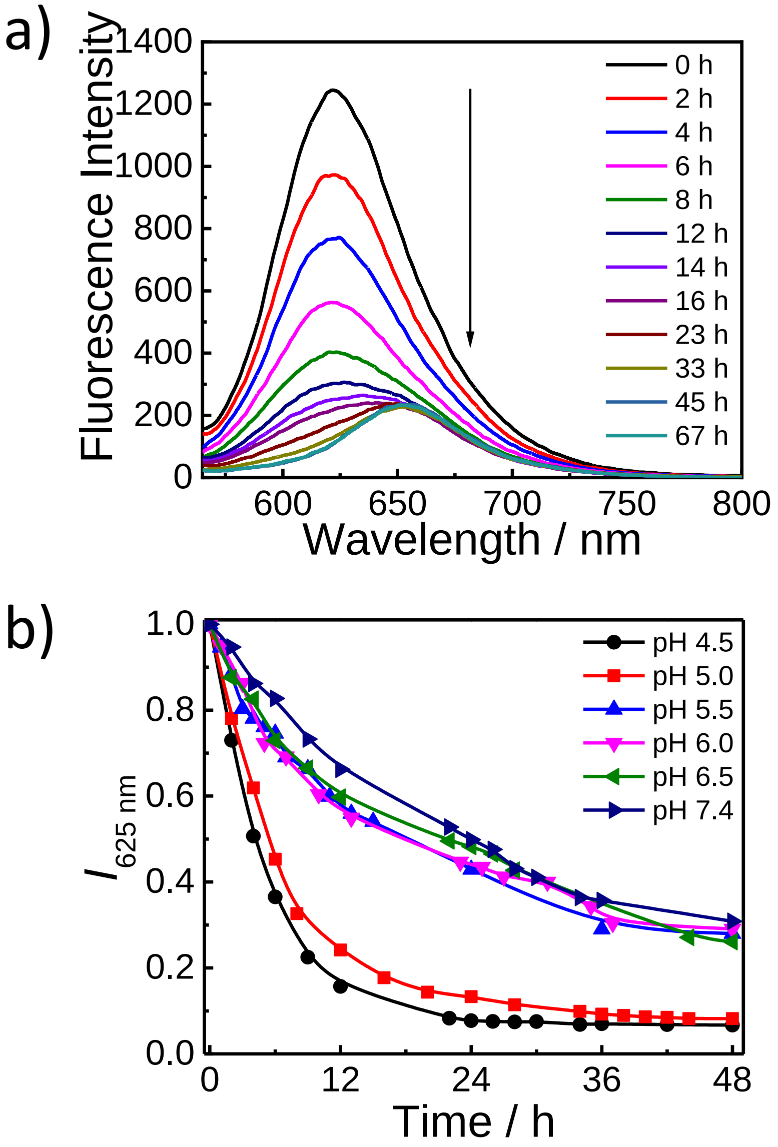

Supplementary Fig. 17 (a) Time-dependent evolution of fluorescence emission spectra (λ_{ex} = 550 nm) and (b) changes in emission intensities at 620 nm recorded for **BPF$_{25}$**-*NR* polymersome dispersions (0.2 g/L) upon incubating at varying pH conditions.

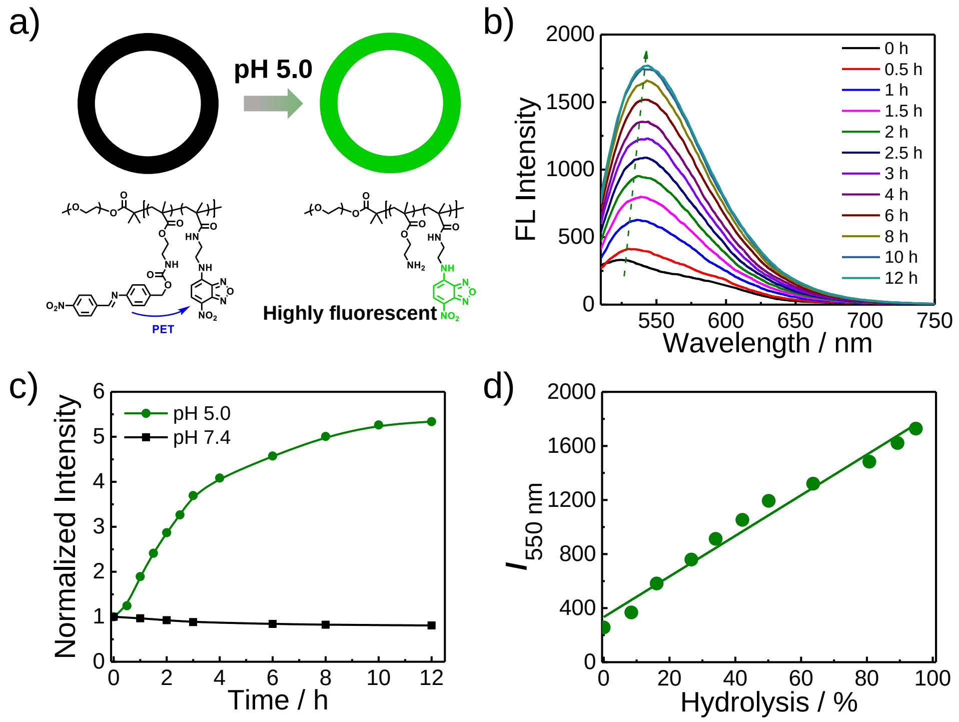

Supplementary Fig. 18 (a) Schematics of fluorogenic **BPN21**-*NBD* polymersomes upon acidic pH-triggered Schiff base hydrolysis and bilayer crosslinking. (b) Time-dependent evolution of fluorescence emission spectra (λ_{ex} = 480 nm) and (c) changes in NBD emission intensities at 550 nm recorded for **BPN21**-*NBD* polymersome dispersions (0.2 g/L) upon incubating at pH 5.0 and pH 7.4, respectively. (d) Correlation of NBD emission intensities at 550 nm ($I_{550\ nm}$) with extents of imine hydrolysis at pH 5.0.

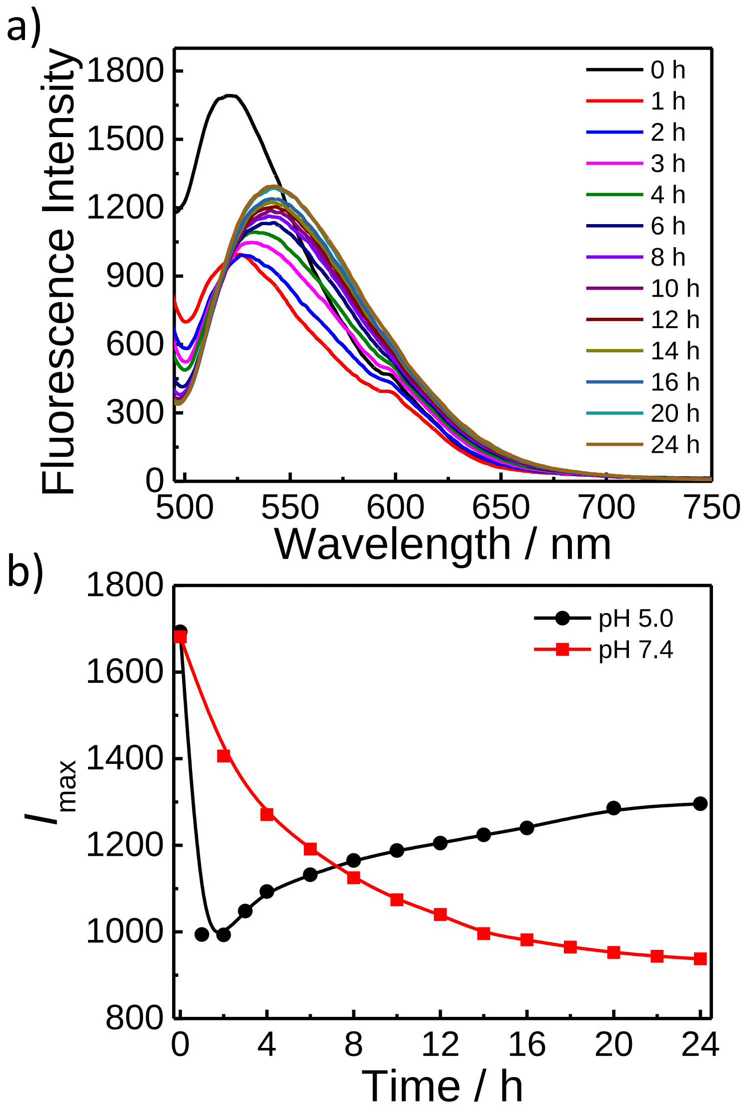

Supplementary Fig. 19 (a) Time-dependent evolution of fluorescence emission spectra (λ_{ex} = 480 nm) recorded for **BPF25-*NBD*** polymersome dispersion (0.2 g/L) upon incubation at pH 5.0. (b) Changes in NBD emission intensities at 550 nm recorded for **BPF25-*NBD*** polymersome dispersions (0.2 g/L) upon incubation at pH 5.0 and pH 7.4, respectively.

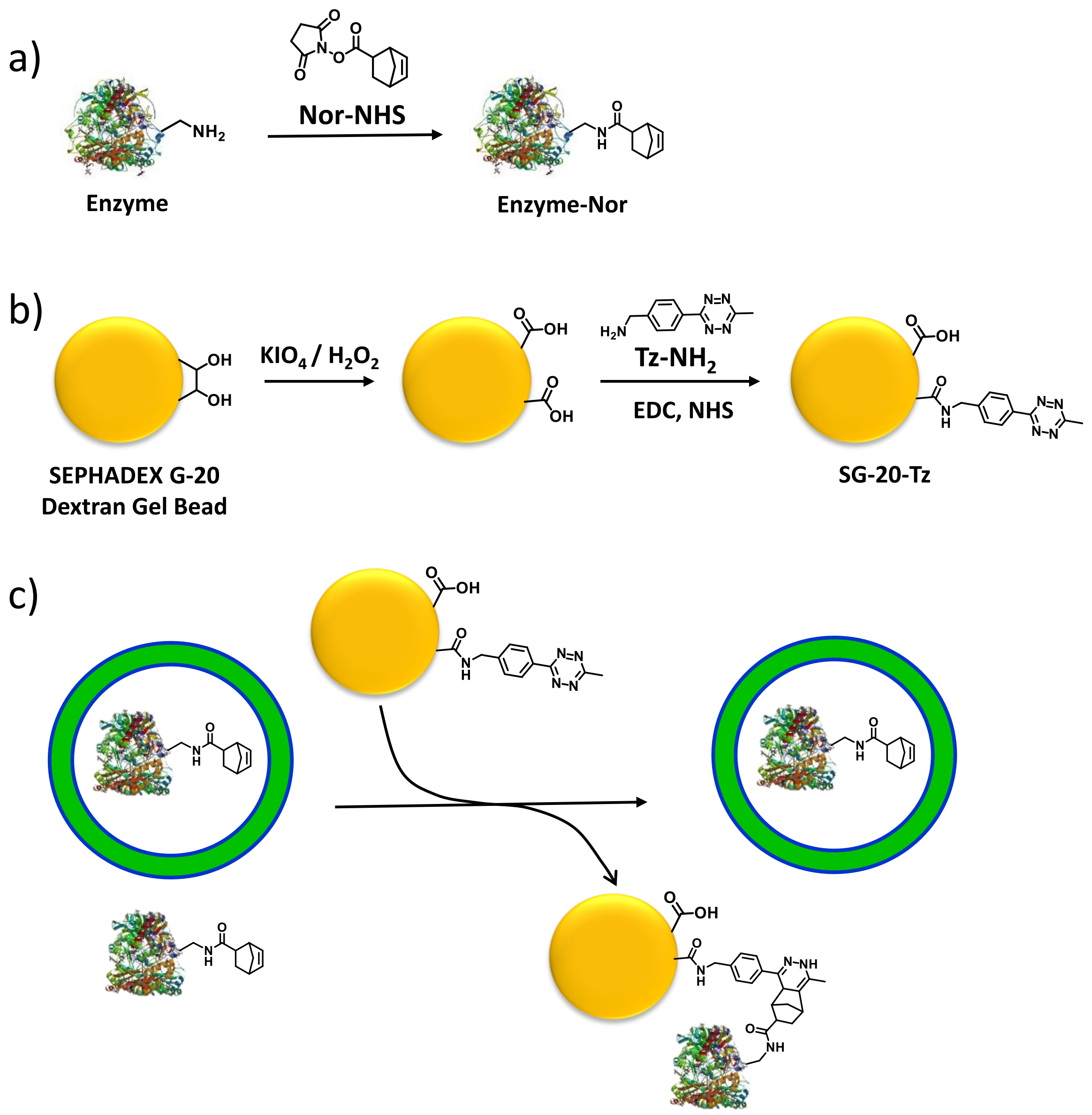

Supplementary Fig. 20 Synthetic routes employed for the fabrication of (a) enzyme-Nor, (b) SG-20-Tz, and (c) removal of free enzyme-Nor using IEDDA reactions on SG-20-Tz gel beads.

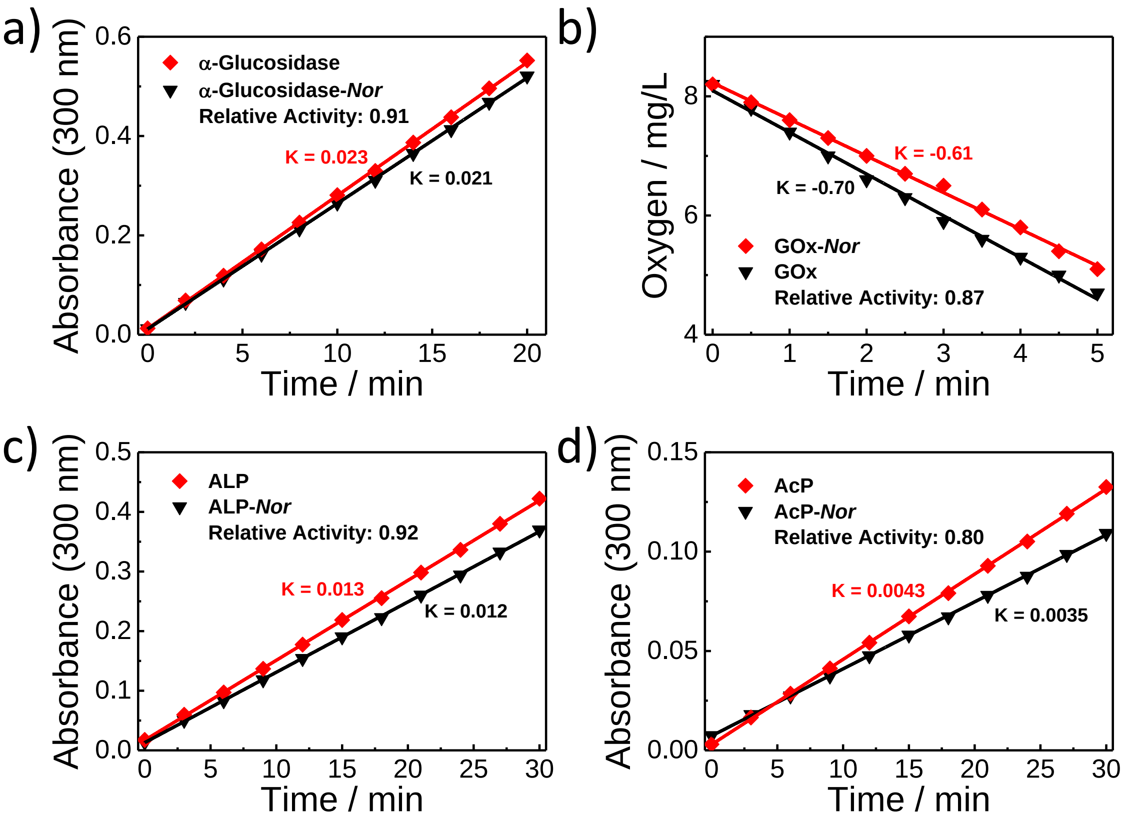

Supplementary Fig. 21 Determination of relative enzyme activity of intact and *Nor*-modified *α*-glucosidase (a), GOx (b), ALP (c), and AcP (d) by scavenging of *p*-nitrophenyl-*β*-D-glucopyranoside (a), oxygen (b), and *p*-nitrophenyl phosphate (c and d), respectively.

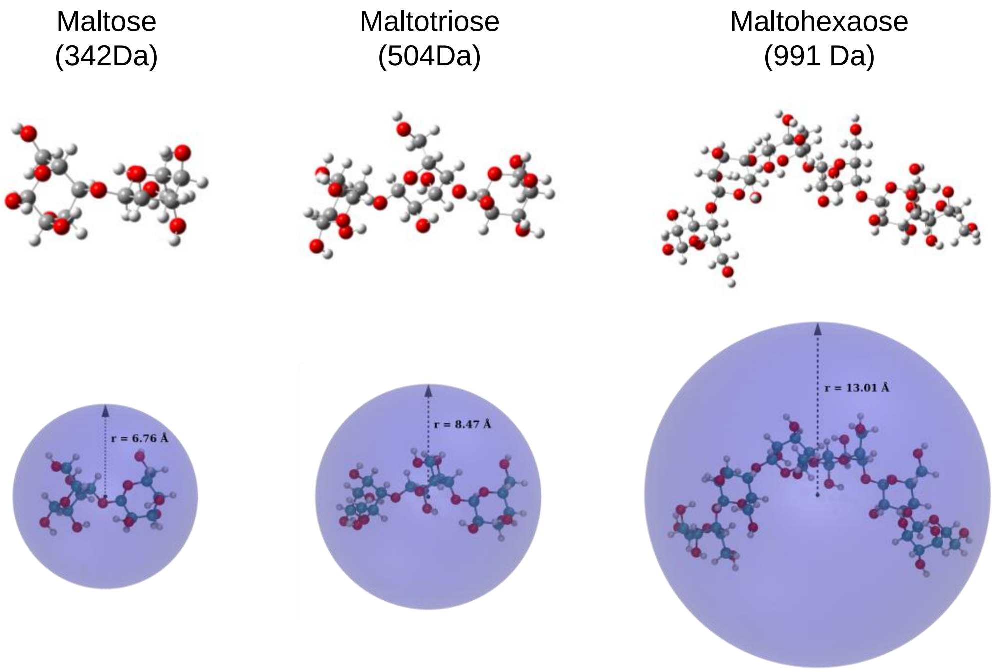

Supplementary Fig. 22 Optimized structures of maltose, maltotriose, and maltohexaose using the density functional theory (DFT) at the level of B3LYP/6-31G(d). The molecular dimensions are calculated by measuring the largest distance of the atoms of each molecule from the geometry center, by adding the Van der Waals radius of hydrogen (1.2 angstrom).

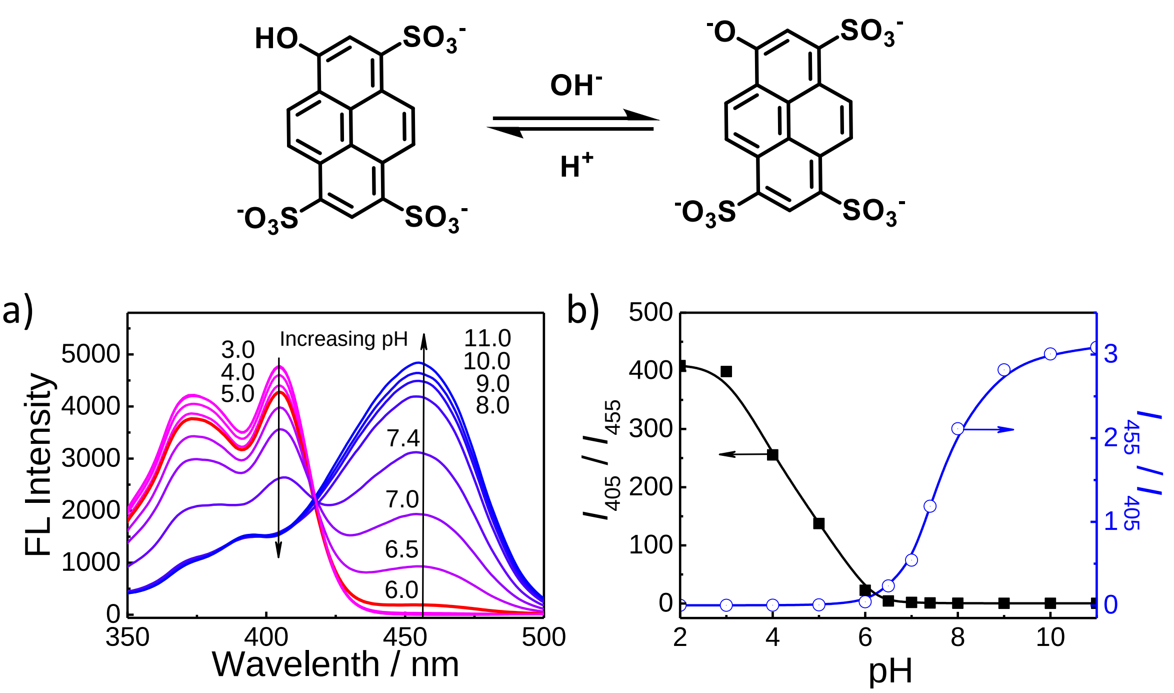

Supplementary Fig. 23 pH-dependent (a) fluorescence excitation spectra (λ_{em} = 510 nm) and (b) excitation intensity ratios, I_{405}/I_{455} and I_{455}/I_{405}, recorded for the aqueous solution of HPTS (20 μM).

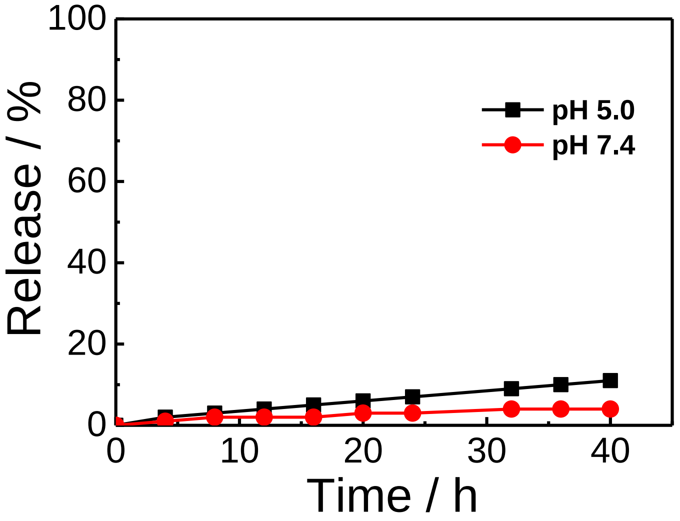

Supplementary Fig. 24 Release profiles of encapsulated HPTS from $\mathbf{BPN_{23}}$ polymersomes (0.2 g/L) upon incubating at pH 7.4 and pH 5.0, respectively.

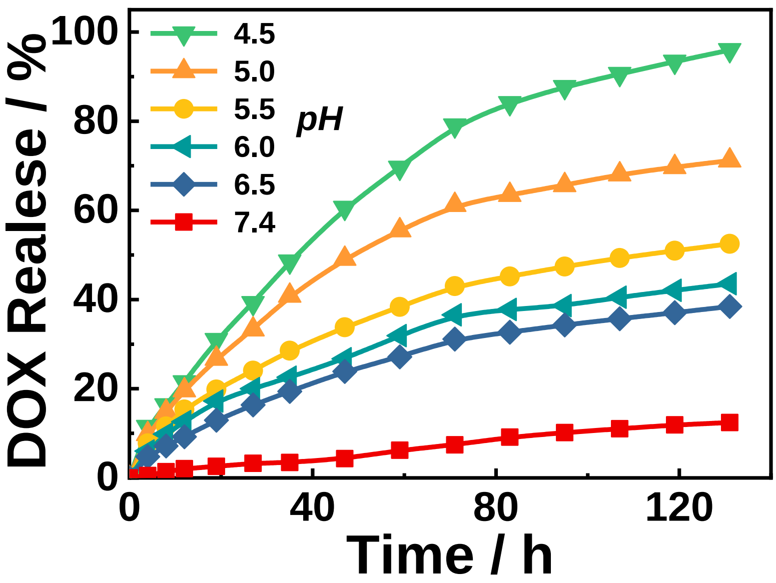

Supplementary Fig. 25 Release profiles of DOX from drug-loaded **BPF$_{25}$** polymersomes (0.2 g/L) upon incubating at varying pH conditions.

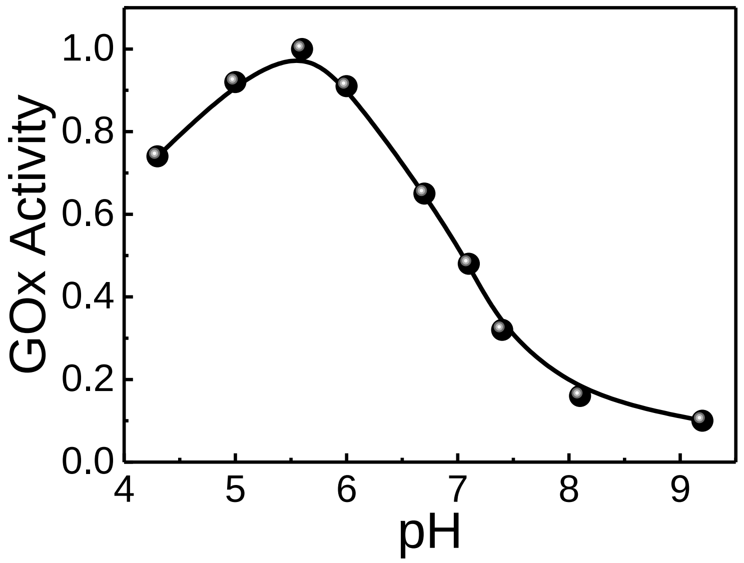

Supplementary Fig. 26 pH-dependent changes of apparent GOx activities assayed by initial oxygen consumption rates upon mixing aqueous solutions of [Ru(dpp)$_3$]Cl$_2$ (1 μM), glucose (5 mM), and GOx (10 mg/L) in buffered medium with pH in the range of 4.4-9.2. The oxygen consumption rates were measured by the evolution of phosphorescence emission intensities at 620 nm (λ_{ex} = 460 nm).

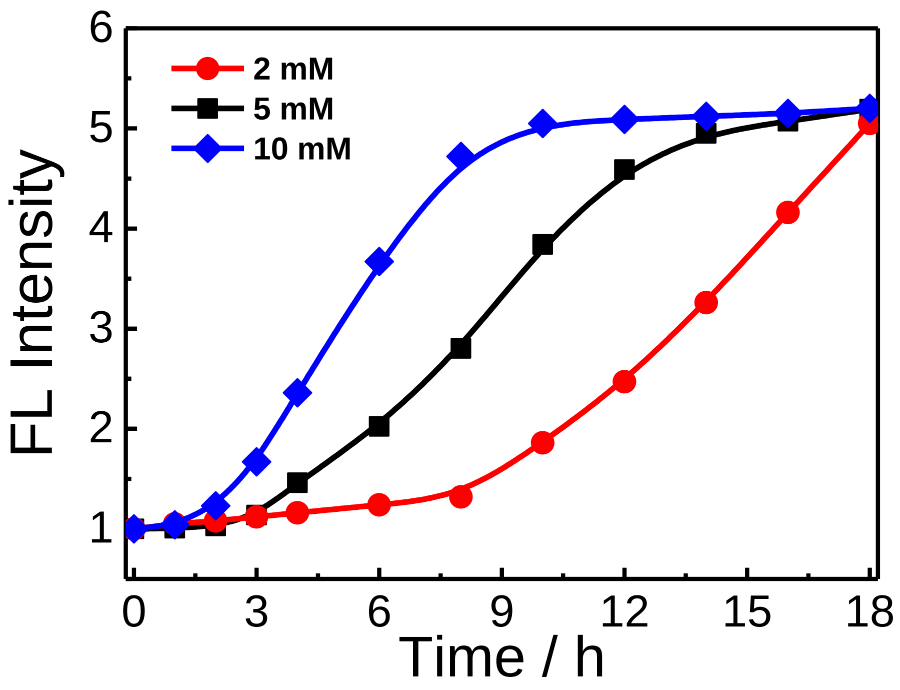

Supplementary Fig. 27 Time-dependent evolution of normalized fluorescence emission intensities (λ_{ex} = 480 nm, λ_{em} = 550 nm) recorded for **BPN$_{21}$-*NBD*** polymersomes encapsulating GOx and Cat enzymes upon addition of glucose (2, 5, and 10 mM); the initial external pH was set at 7.4.

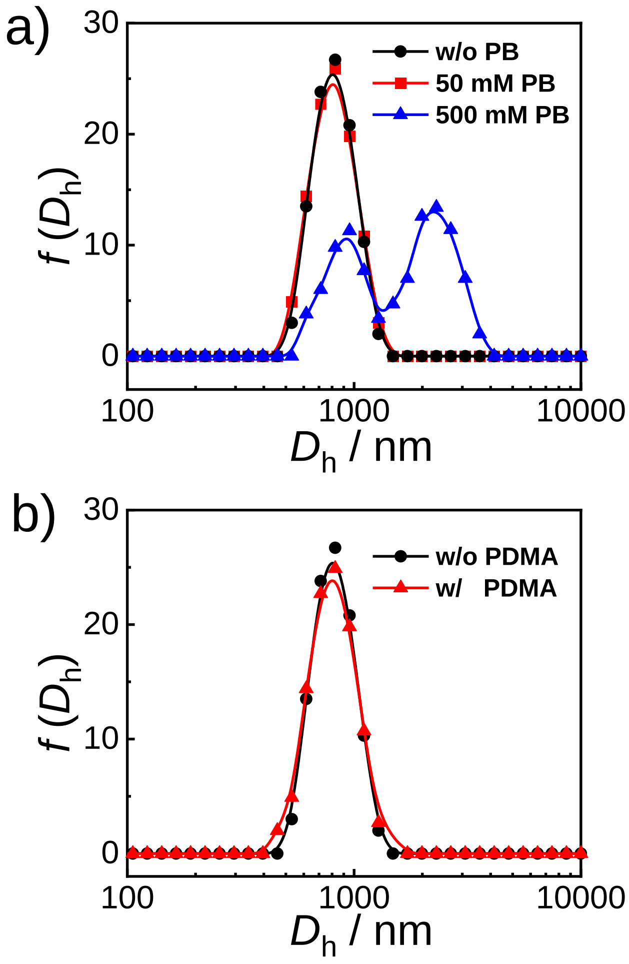

Supplementary Fig. 28 Hydrodynamic diameter distributions, $f(D_h)$, recorded for **BPN$_{23}$** polymersomes in the presence of (a) external PB buffer (pH 7.4, 50 or 500 mM) and (b) PDMA (1.57 g/L).

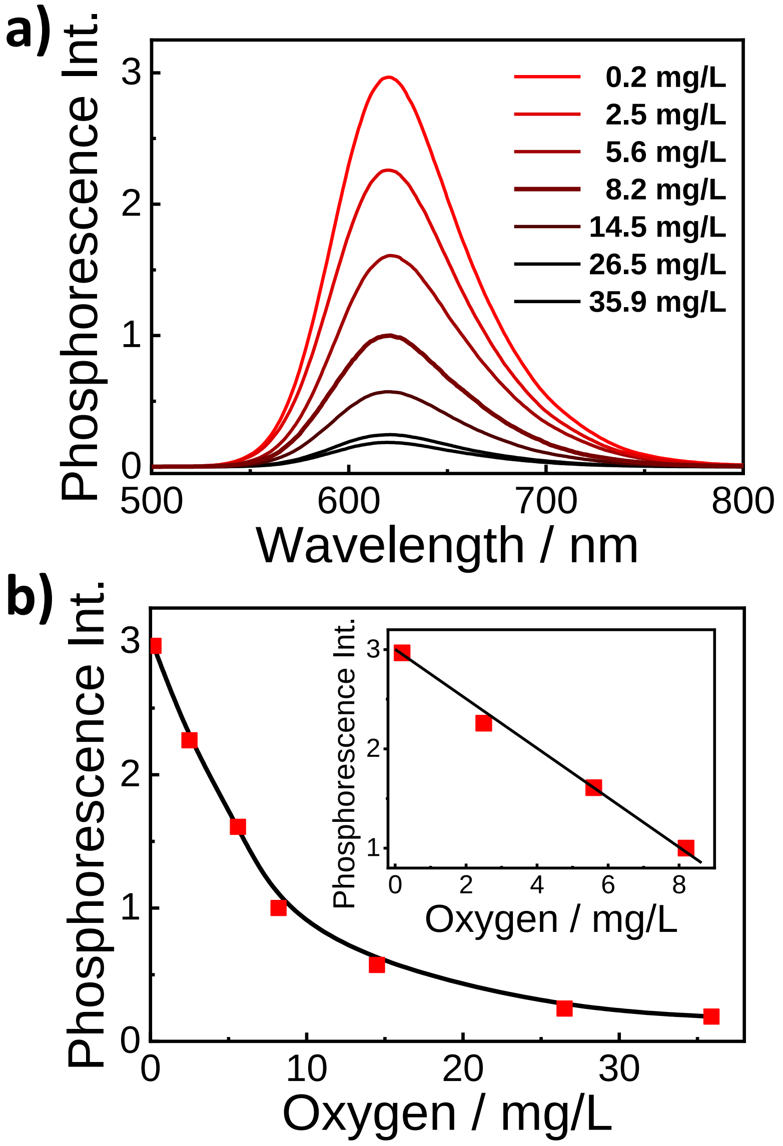

Supplementary Fig. 29 Oxygen level-dependent (a) phosphorescence emission spectra and (b) changes in emission intensities at 620 nm (λ_{ex} = 460 nm) recorded for an aqueous solution of $[Ru(dpp)_3]Cl_2$ (1.0 μM).

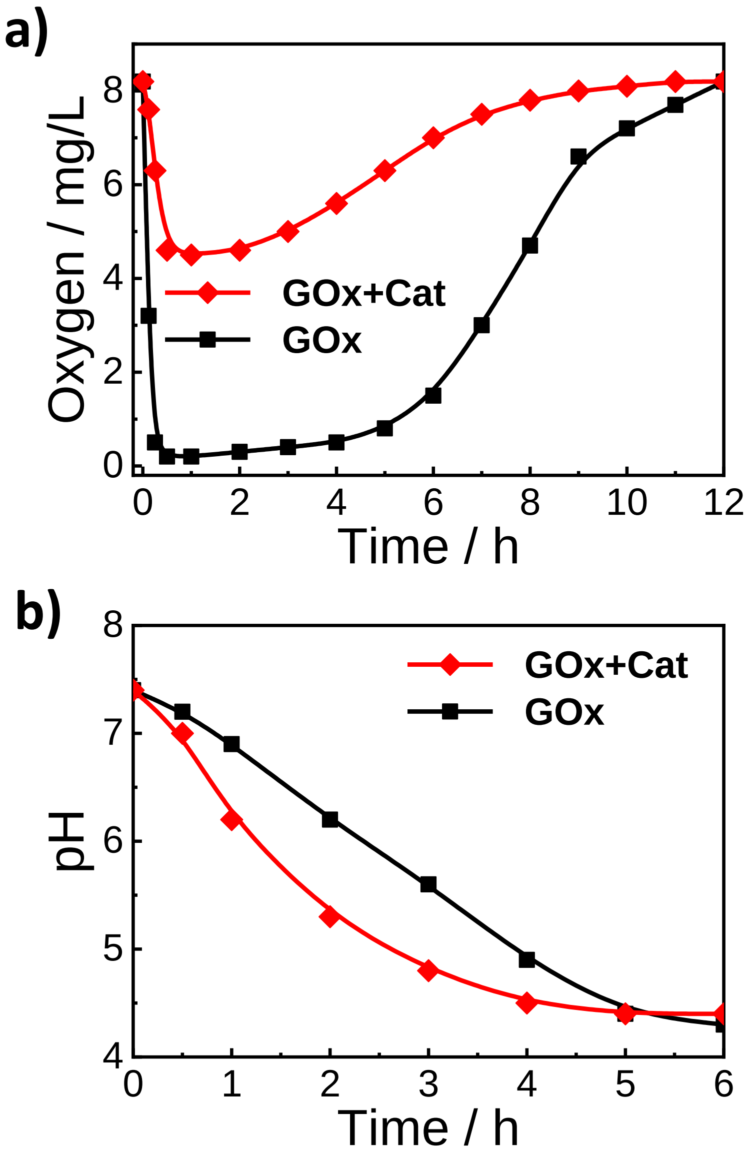

Supplementary Fig. 30 (a) Incubation time-dependent evolution of oxygen levels upon mixing [Ru(dpp)$_3$]Cl$_2$ (1 μM), glucose (5 mM), and GOx (10 mg/L) solutions (20 mM acetate buffer, pH 5.6) in the absence and presence of Cat (1 mg/L). (b) Incubation time-dependent evolution of solution pH upon mixing HPTS (10 μM), glucose (5 mM), and GOx (10 mg/L) in the absence and presence of Cat (1 mg/L).

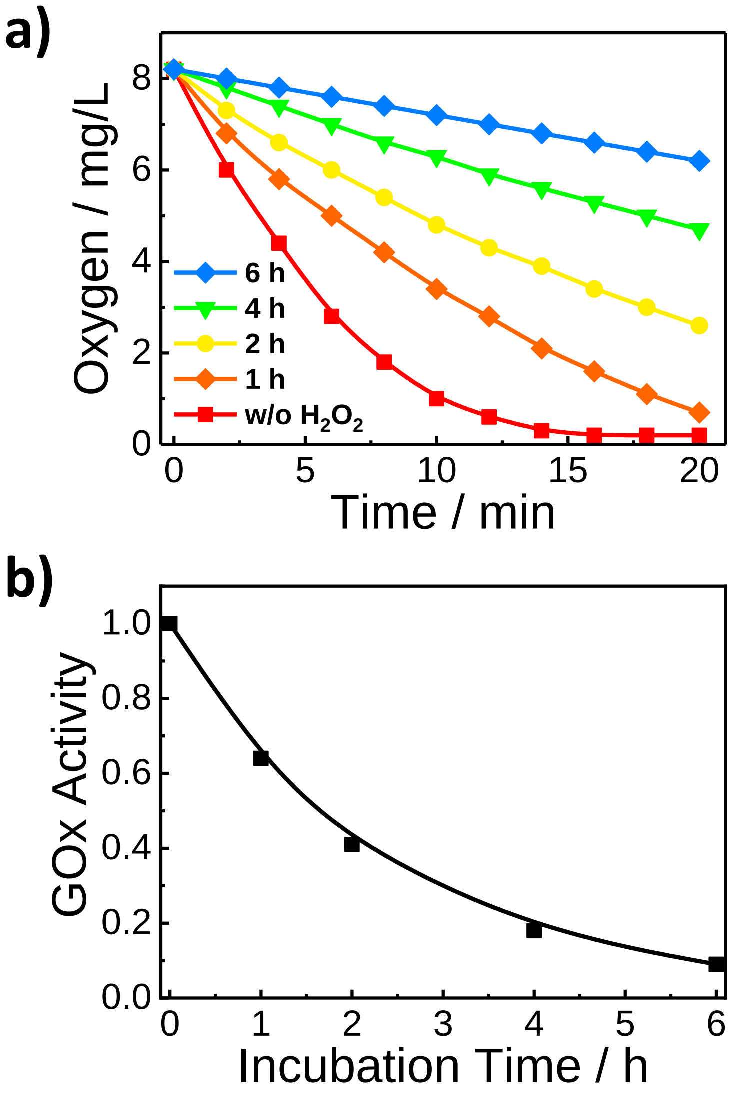

Supplementary Fig. 31 (a) Time-dependent evolution of oxygen levels upon mixing [Ru(dpp)$_3$]Cl$_2$ (1 μM), glucose (5 mM), and GOx (10 mg/L) solutions (20 mM acetate buffer, pH 5.6); before mixing, the GOx solution was pre-treated with H_2O_2 for varying durations (1-6 h). (b) H_2O_2 incubation duration-dependent relative GOx activities assayed by initial oxygen consumption rates. The oxygen consumption rates were measured by the evolution of phosphorescence emission intensities at 620 nm (λ_{ex} = 460 nm).

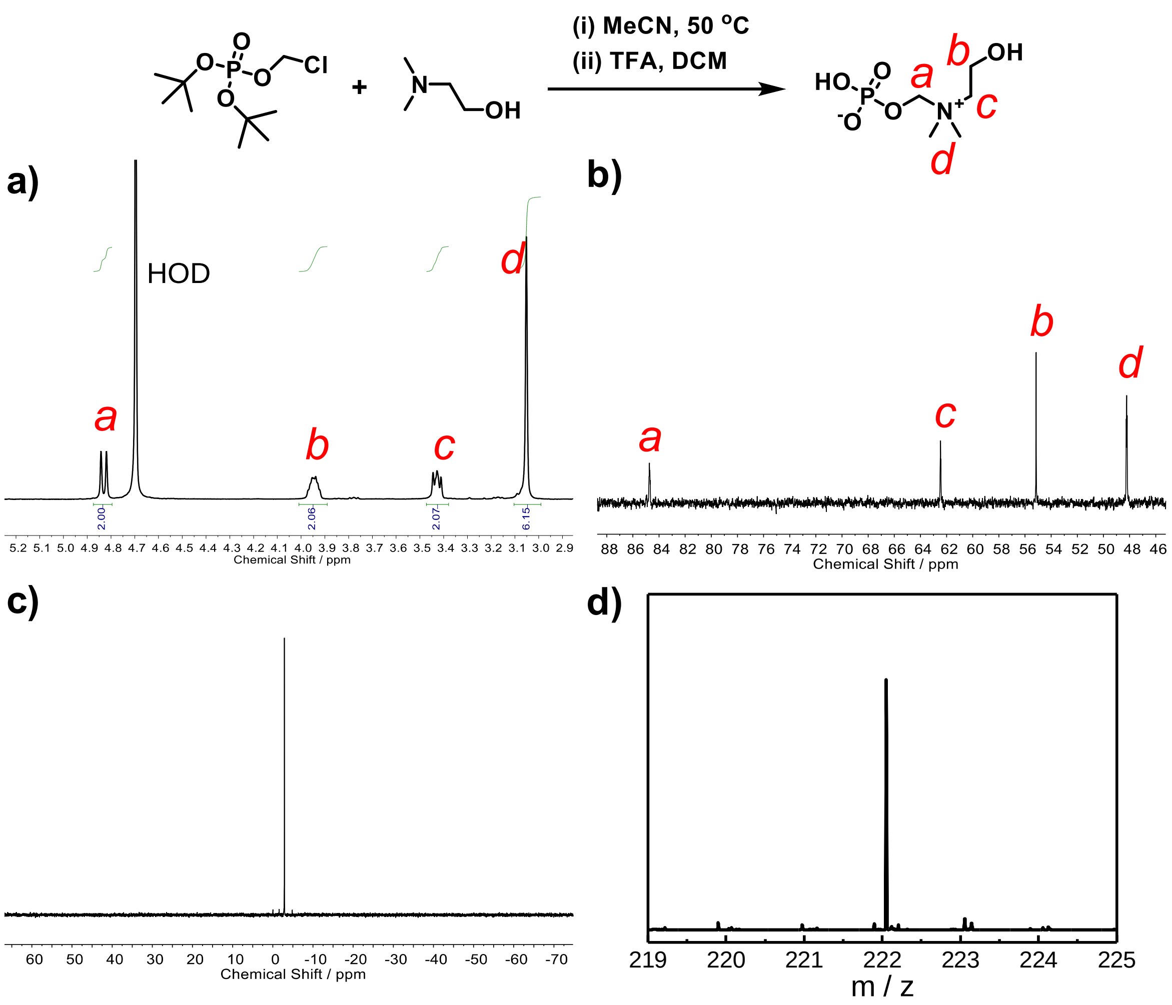

Supplementary Fig. 32 Synthetic scheme employed for the preparation of zwitterionic PMN and corresponding ^{1}H NMR (a), ^{13}C NMR (b), and ^{31}P NMR (c) spectra recorded in D_2O and ESI mass spectrum (d).

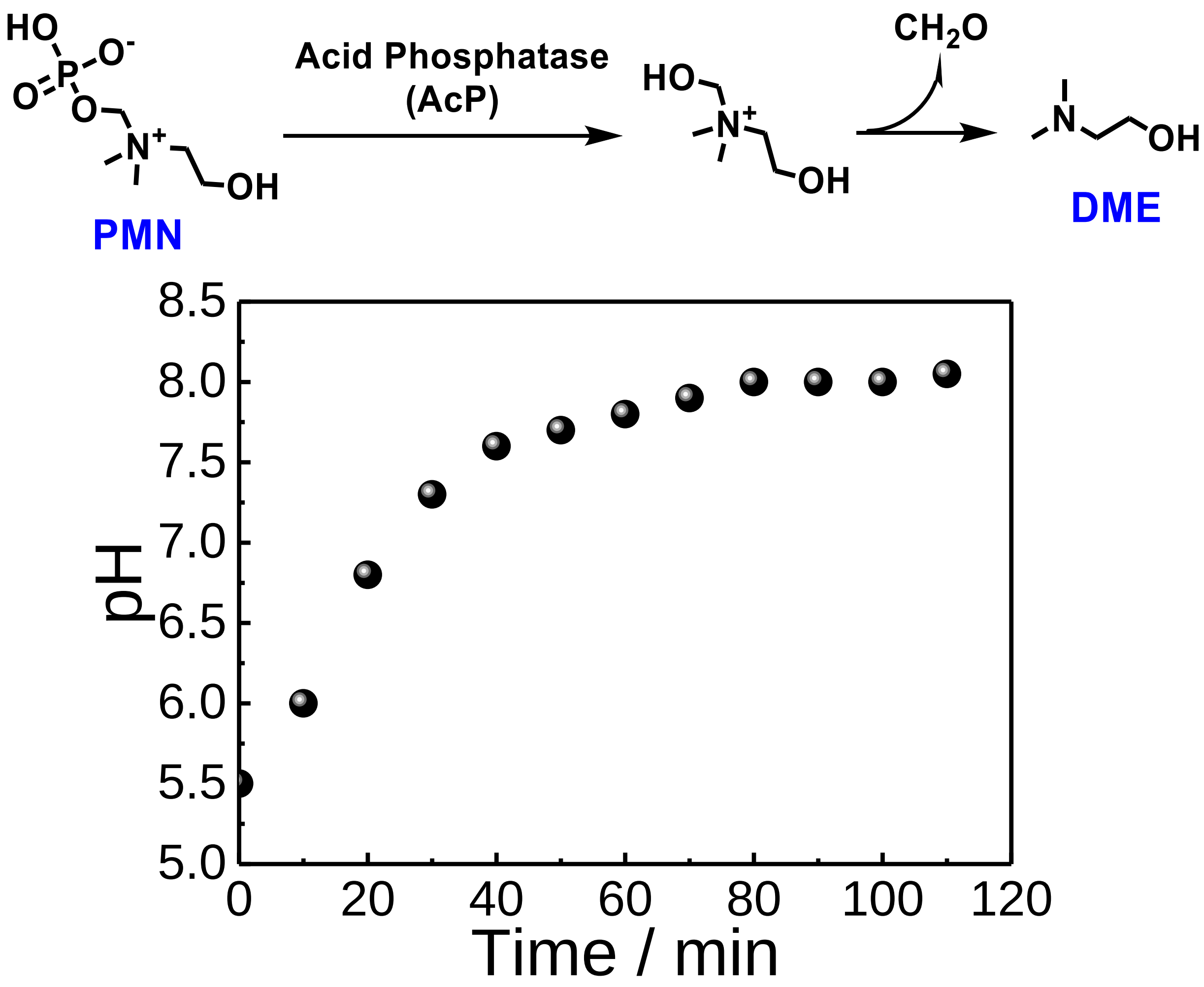

Supplementary Fig. 33 Time-dependent evolution of pH recorded for the aqueous PMN solution (12 mM) upon mixing with AcP enzyme (5.0 mg/L); the initial solution pH was set at 5.5.

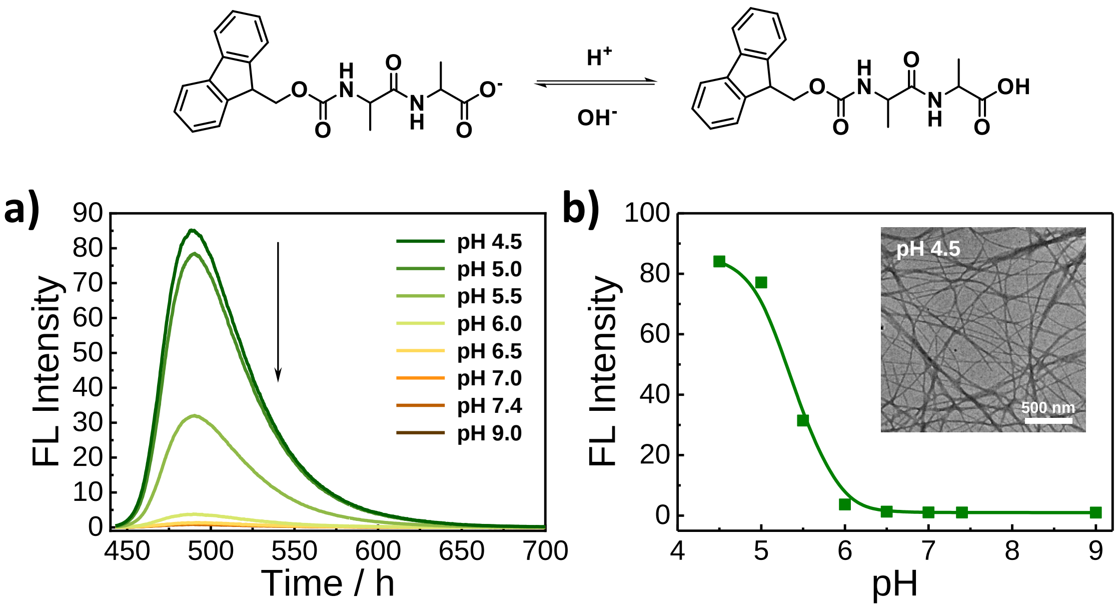

Supplementary Fig. 34 pH-dependent evolution of (a) fluorescence emission spectra (λ_{ex} = 420 nm) and (b) emission intensities (λ_{em} = 490 nm) recorded for the aqueous solution of Fmoc-AA-OH (3.6 mg/mL) in the presence of ThT probe (1 μM).

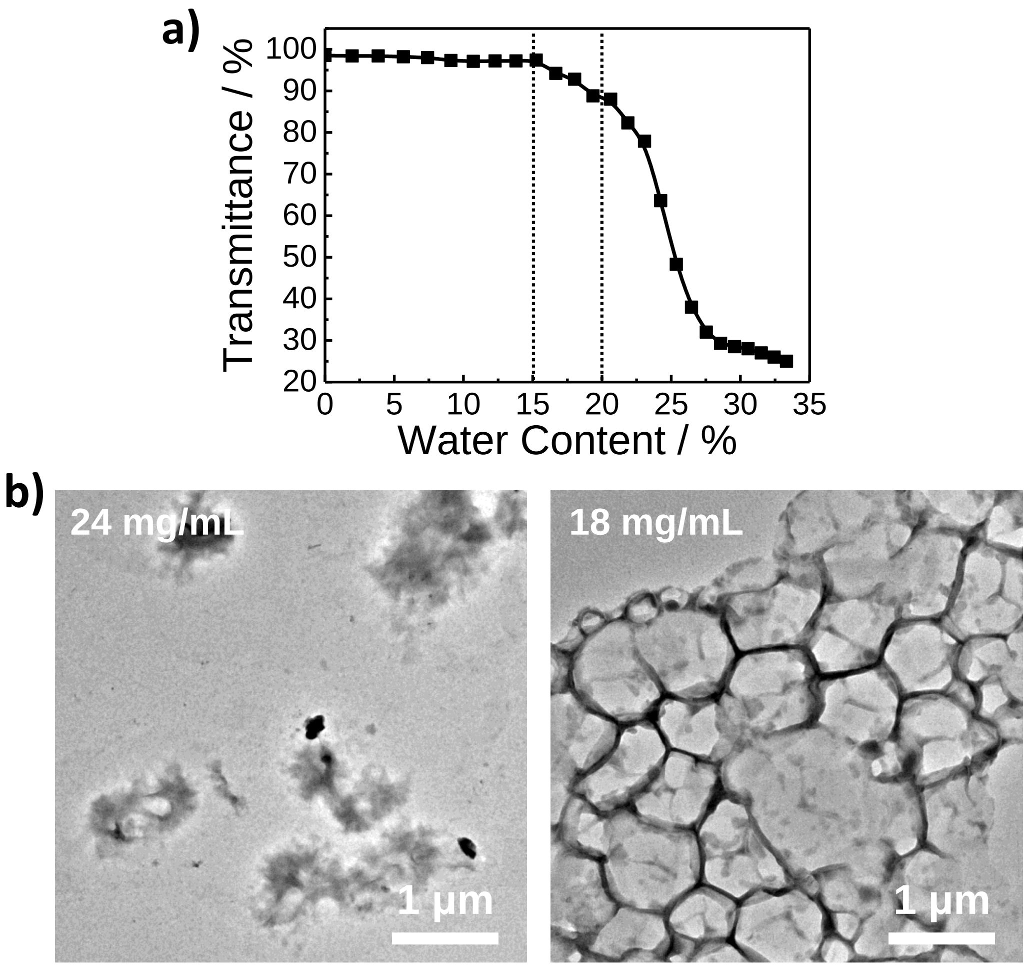

Supplementary Fig. 35 (a) Variation of optical transmittance at 800 nm as a function of water contents (v/v) upon water addition into **BPN$_{23}$** solution (2.0 g/L) in 1,4-dioxane. (b) TEM images of nanostructures self-assembled from **BPN$_{23}$**; the self-assembly was actuated by addition of 1.0 mL aqueous solution of Fmoc-AA-OH (pH 9.0; 24 mg/mL or 18 mg/mL) into **BPN$_{23}$** solution (2.0 g/L) in 1,4-dioxane (1 mL), followed by the addition of 8.0 mL deionized water (pH 9.0). The addition rates were set at 1.0 mL/h in all cases.

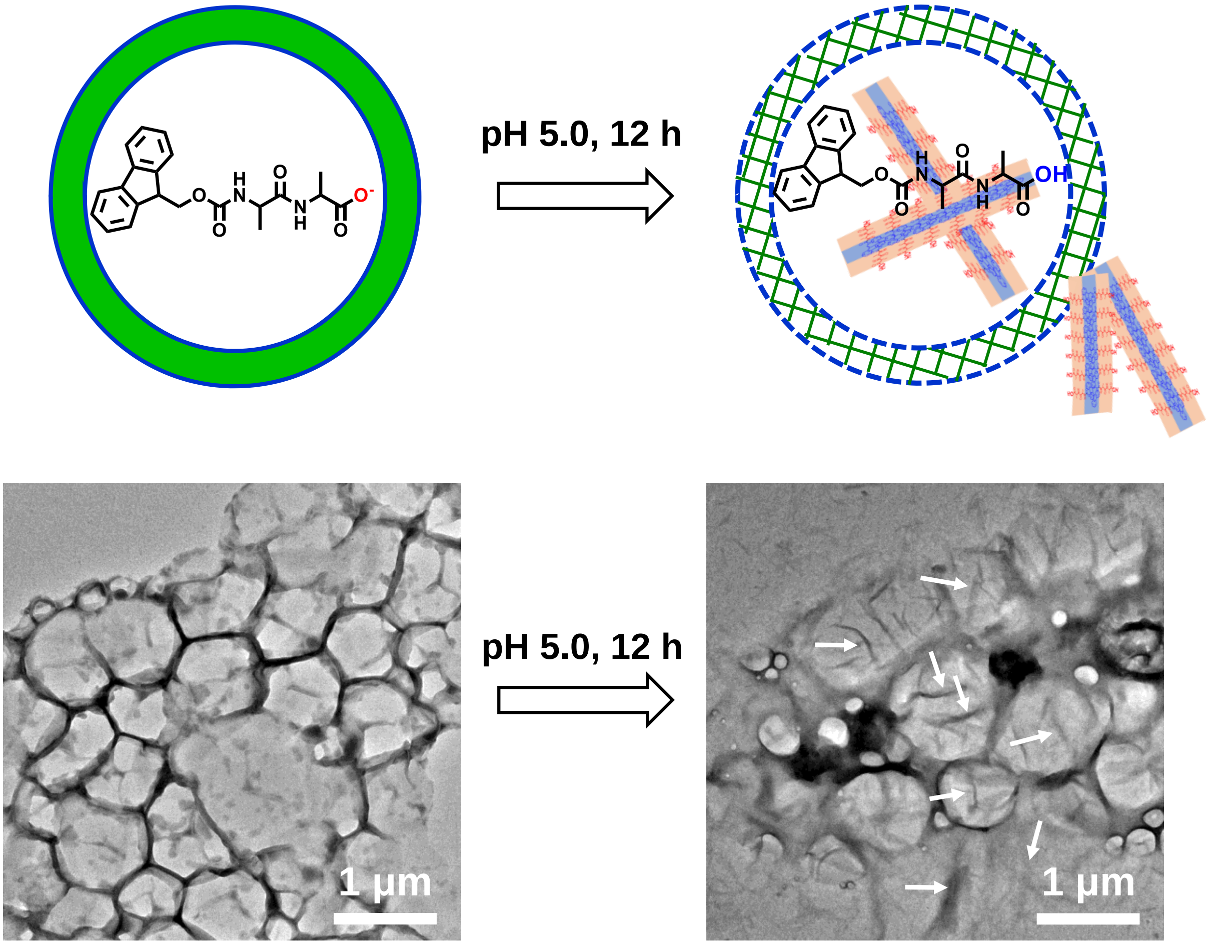

Supplementary Fig. 36 TEM images recorded for **BPN$_{23}$** polymersomes encapsulating Fmoc-AA-OH dipeptide before and after incubation at pH 5.0 for 12 h. The white arrows indicate the as-assembled nanofibers of Fmoc-AA-OH dipeptide.

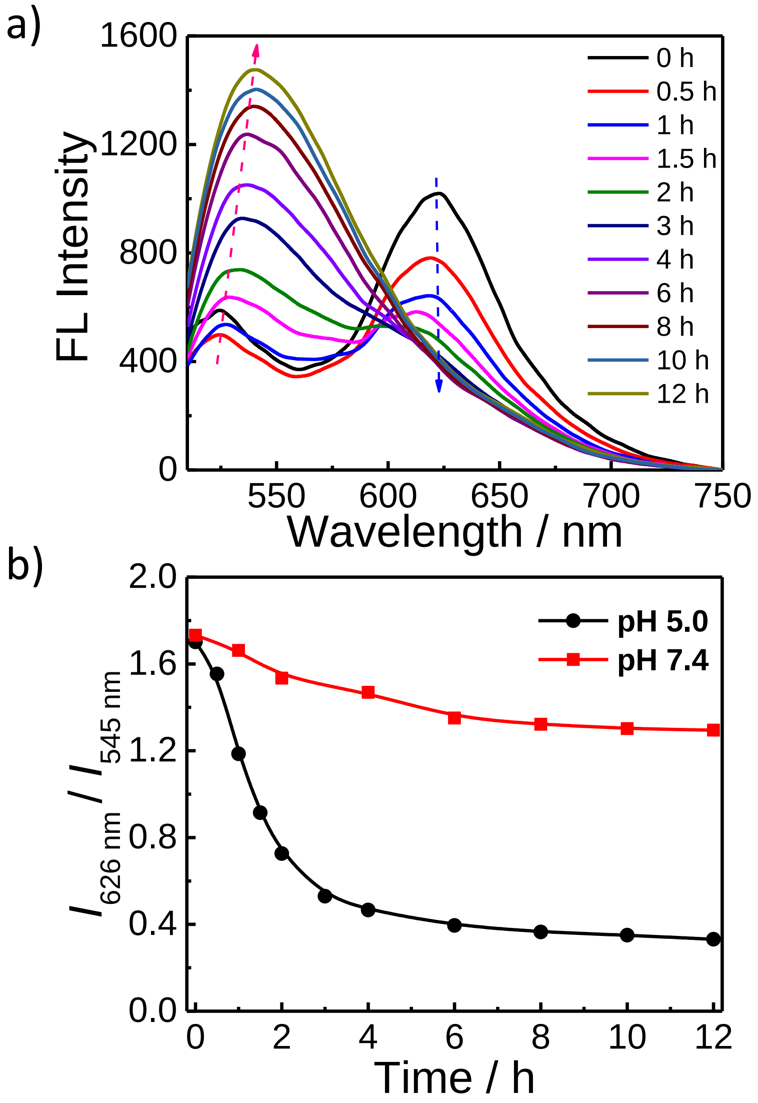

Supplementary Fig. 37 (a) Time-dependent evolution of fluorescence emission spectra (λ_{ex} = 480 nm) recorded for **BPN$_{21}$**-*NBD*/**BPN$_{24}$**-*NR* (7/3 wt/wt) co-assembled polymersome dispersion upon incubation at pH 5.0. (b) Time-dependent evolution of emission intensity ratios, $I_{626\ nm}/I_{545\ nm}$, recorded for **BPN$_{21}$**-*NBD*/**BPN$_{24}$**-*NR* (7/3 wt/wt) co-assembled polymersome dispersion upon incubation at pH 5.0 and pH 7.4, respectively.

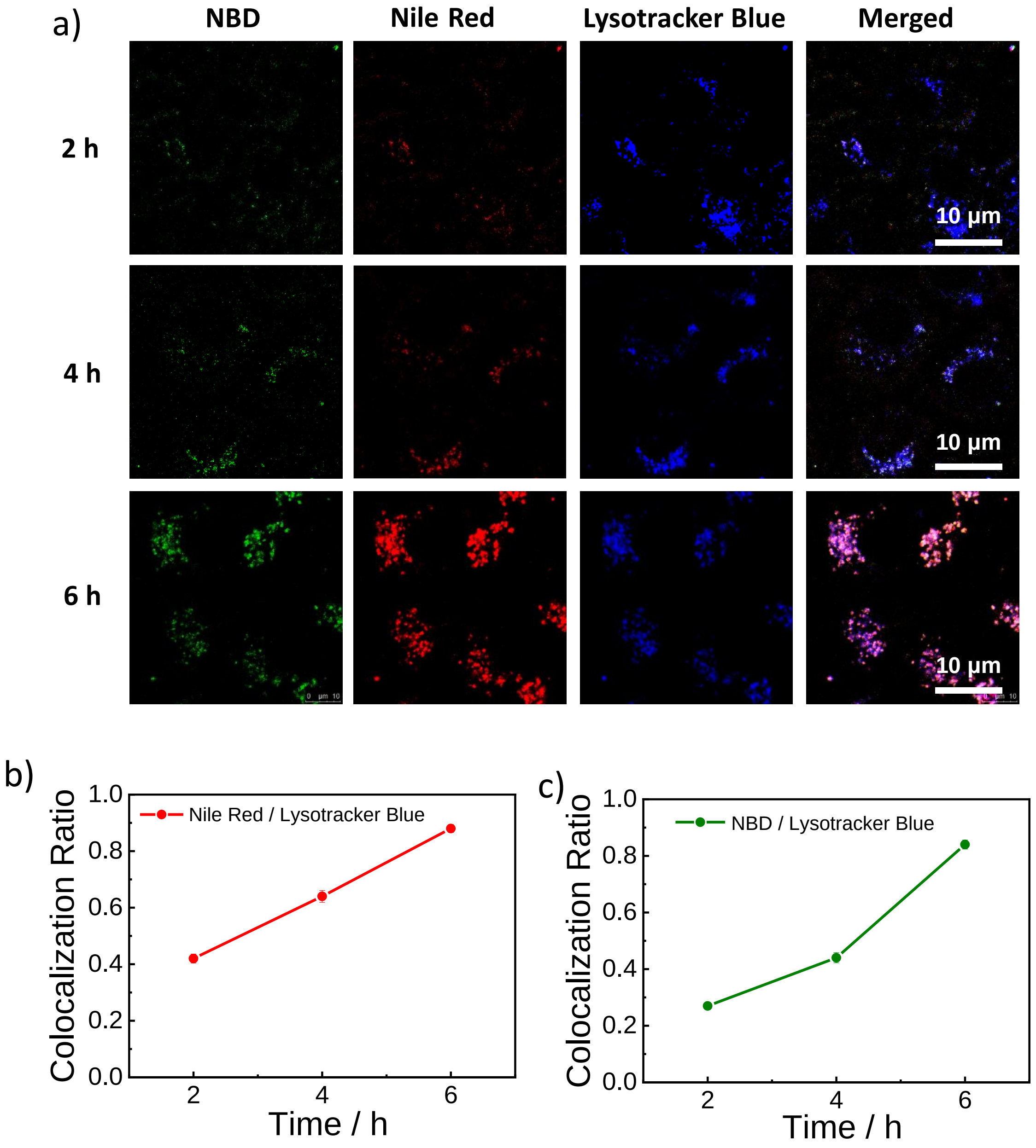

Supplementary Fig. 38 (a) CLSM images of HepG2 cells recorded after co-incubating with **BPN**$_{21}$**-***NBD*/**BPN**$_{24}$**-***NR* (7/3 wt./wt., 0.2 g/L) co-assembled polymersome dispersions for varying durations. Time-dependent evolution of colocalization ratios between red channel (*NR*-vesicles) and blue channel images (b), and between green channel (*NBD*-vesicles) and blue channel images (c) quantified from CLSM results. Data are presented as mean ± SD (n = 3). The endolysosomes were stained with Lysotracker Blue (blue channel). The green channel was excited at 488 nm and collected between 510-540 nm; the red channel was excited at 543 nm and collected between 555-595 nm; the blue channel was excited at 405 nm and collected between 415-475 nm. Error bars represent mean ± SD (n = 10 independent cells).

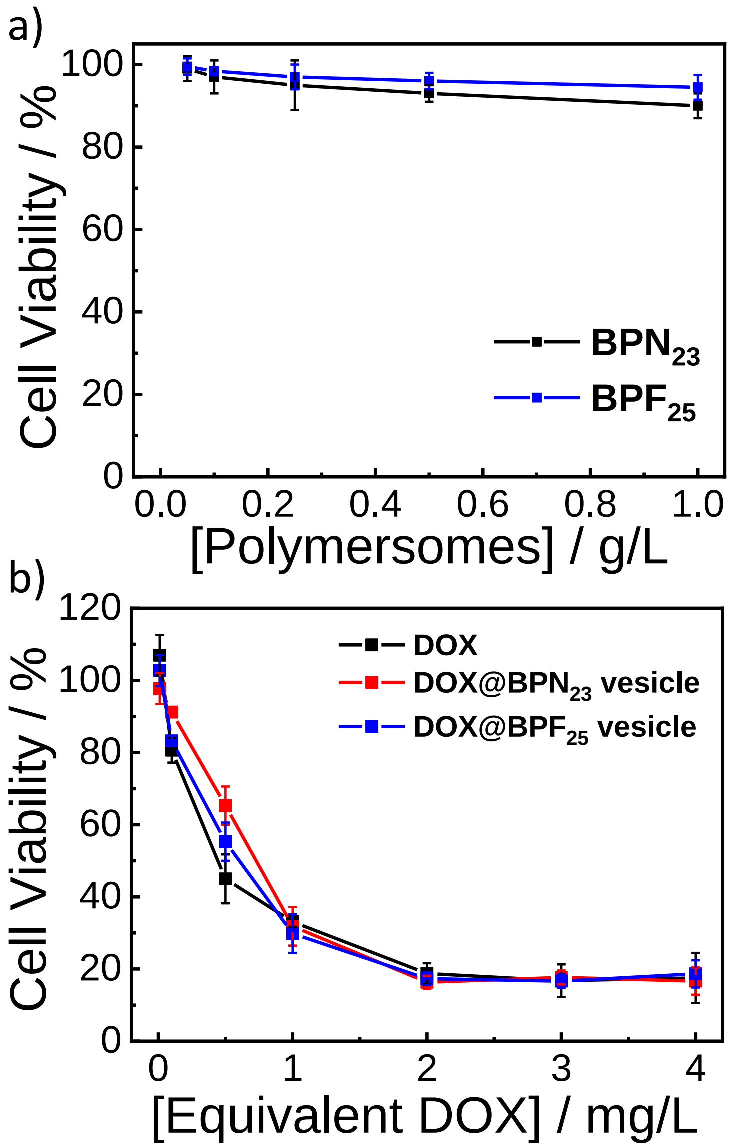

Supplementary Fig. 39 (a) *In vitro* cytotoxicity assay against HepG2 cells after 48 h co-incubation with **BPN23** and **BPF25** polymersome dispersions of varying concentrations. (b) Cellular proliferation inhibition of DOX-loaded **BPN23** and **BPF25** polymersome dispersions and free DOX toward HepG2 cells as a function of equivalent DOX concentrations. Results are presented as means ± SD in quintuplicate.
