# Supplementary material for: Oscillating the local milieu of polymersome interiors via single input-regulated bilayer crosslinking and permeability tuning
Source: Nat Commun. 2022 Jan 31;13:585. doi: 10.1038/s41467-022-28227-6 (PMC8803951; doi:10.1038/s41467-022-28227-6)
Supplement: Supplementary file 2 — Reporting Summary [file 41467_2022_28227_MOESM2_ESM.pdf]

nature portfolio

Corresponding author(s): Jinming Hu and Shiyong Liu

Last updated by author(s): Dec 15, 2021

Reporting Summary

Nature Portfolio wishes to improve the reproducibility of the work that we publish. This form provides structure for consistency and transparency in reporting. For further information on Nature Portfolio policies, see our Editorial Policies and the Editorial Policy Checklist.

Statistics

For all statistical analyses, confirm that the following items are present in the figure legend, table legend, main text, or Methods section.

n/a	Confirmed	
☐	☒	The exact sample size (*n*) for each experimental group/condition, given as a discrete number and unit of measurement
☐	☒	A statement on whether measurements were taken from distinct samples or whether the same sample was measured repeatedly
☒	☐	The statistical test(s) used AND whether they are one- or two-sided<br>*Only common tests should be described solely by name; describe more complex techniques in the Methods section.*
☒	☐	A description of all covariates tested
☒	☐	A description of any assumptions or corrections, such as tests of normality and adjustment for multiple comparisons
☐	☒	A full description of the statistical parameters including central tendency (e.g. means) or other basic estimates (e.g. regression coefficient) AND variation (e.g. standard deviation) or associated estimates of uncertainty (e.g. confidence intervals)
☒	☐	For null hypothesis testing, the test statistic (e.g. *F*, *t*, *r*) with confidence intervals, effect sizes, degrees of freedom and *P* value noted<br>*Give P values as exact values whenever suitable.*
☒	☐	For Bayesian analysis, information on the choice of priors and Markov chain Monte Carlo settings
☒	☐	For hierarchical and complex designs, identification of the appropriate level for tests and full reporting of outcomes
☒	☐	Estimates of effect sizes (e.g. Cohen's *d*, Pearson's *r*), indicating how they were calculated

Our web collection on statistics for biologists contains articles on many of the points above.

Software and code

Policy information about availability of computer code

Data collection	Wyatt GPC instrument was operated with ASTRA (version: 6.1); Bruker AV300 NMR was operated with XWIN- NMR (version: 3.1); ALV/DLS/SLS-5022F DLS spectrometer was operated with ALV-7004 (V.3.0.5.2); Leica TCS SP5 microscope was operated with LAS AF (version: 2.6.0); Hitachi F-4600 fluorescence spectrophotometer was operated with FL Solution for F4600 (version 2.1); Shimadzu UV-3600 Plus spectrophotometer was operated with UVWin (version 5.0).
Data analysis	The data analyses were performed on Origin (version 2020), Excel 2019, MestReNova (14.2.0), Image-pro plus (6.0.0) and Image J (version 1.52a) .

For manuscripts utilizing custom algorithms or software that are central to the research but not yet described in published literature, software must be made available to editors and reviewers. We strongly encourage code deposition in a community repository (e.g. GitHub). See the Nature Portfolio guidelines for submitting code & software for further information.

Data

Policy information about availability of data

All manuscripts must include a data availability statement. This statement should provide the following information, where applicable:

- Accession codes, unique identifiers, or web links for publicly available datasets
- A description of any restrictions on data availability
- For clinical datasets or third party data, please ensure that the statement adheres to our policy

The main data in this study are available within the manuscript and its supplementary information. The raw data are available from the corresponding authors on reasonable request. The source data underlying Figs. 2a-d, h-j, 3c, d, f, g, 4b–j, 5b, e, 6b, d, and Supplementary Figs. 2-6, 9d, 10-13, 14a, b, 15, 16c-e, 17-19, 21,

23-34, 35a, 37, 38b, c, and 39 are provided as a Source Data file.

Field-specific reporting

Please select the one below that is the best fit for your research. If you are not sure, read the appropriate sections before making your selection.

[x] Life sciences [ ] Behavioural & social sciences [ ] Ecological, evolutionary & environmental sciences

For a reference copy of the document with all sections, see nature.com/documents/nr-reporting-summary-flat.pdf

Life sciences study design

All studies must disclose on these points even when the disclosure is negative.

Sample size	No sample size calculation was performed in advance. Samples size are determined according to comparable experiments in previous studies.
Data exclusions	No data were excluded.
Replication	Experiments were repeated and experimental findings were reproducible. Details of experimental procedures are given in the figure legends or methods.
Randomization	Randomization was not necessary for in vitro cell viability studies because cells from the same culture dish were used for different groups within each experiment to keep the same experimental conditions.
Blinding	In vitro cell experiments was not blinded, as the experiments were performed by the same investigator.

Reporting for specific materials, systems and methods

We require information from authors about some types of materials, experimental systems and methods used in many studies. Here, indicate whether each material, system or method listed is relevant to your study. If you are not sure if a list item applies to your research, read the appropriate section before selecting a response.

Materials & experimental systems

n/a	Involved in the study	
[x]	[ ]	Antibodies
[ ]	[x]	Eukaryotic cell lines
[x]	[ ]	Palaeontology and archaeology
[x]	[ ]	Animals and other organisms
[x]	[ ]	Human research participants
[x]	[ ]	Clinical data
[x]	[ ]	Dual use research of concern

Methods

n/a	Involved in the study	
[x]	[ ]	ChIP-seq
[x]	[ ]	Flow cytometry
[x]	[ ]	MRI-based neuroimaging

Eukaryotic cell lines

Policy information about cell lines

Cell line source(s)	The human hepatocarcinoma cell line (HepG2).
Authentication	The used cell line was procured from Cell Bank of the Chinese Academy of Sciences (Shanghai, China) and not authenticated.
Mycoplasma contamination	The used cell line was tested as negative for mycoplasma contamination.
Commonly misidentified lines (See ICLAC register)	No commonly misidentified lines were used in this study.
